# On-chip photodetection of angular momentums of vortex structured light

**DOI:** 10.1038/s41467-024-49855-0

**Published:** 2024-06-26

**Authors:** Mingjin Dai, Chongwu Wang, Fangyuan Sun, Qi Jie Wang

**Affiliations:** 1 School of Electrical and Electronic Engineering, Nanyang Technological University, Singapore, 639798 Singapore; 2 Centre for Disruptive Photonic Technologies, School of Physical and Mathematical Sciences, Nanyang Technological University, Singapore, 637371 Singapore

**Keywords:** Mid-infrared photonics, Nanophotonics and plasmonics

## Abstract

Structured vortex light with orbital angular momentum (OAM) shows great promise for high-bandwidth optical communications, quantum information and computing, optical tweezers, microscopy, astronomy, among others. Generating, controlling, and detecting of vortex light by all-electrical means is at the heart of next generation nanophotonic platforms. However, on-chip electrical photodetection of structured vortex light remains challenging. Here, we propose an on-chip photodetector based on 2D broadband thermoelectric material (PdSe_2_) with a well-designed spin-Hall couplers to directly characterize angular momentum modes of vortex structured light. Photothermoelectric responses in the PdSe_2_ nanoflake, excited by the focusing surface plasmons, show a magnitude proportional to the total angular momentum modes of the infrared vortex beams, thereby achieving direct detection of spin and orbital angular momentum, as well as the chirality and ellipticity of scalar vortex lights. Our works provide a promising strategy for developing on-chip angular momentum optoelectronic devices, which play a key role in the next-generation high-capacity optical communications, quantum information and computing, imaging, and other photonic systems.

## Abstract (web summary)

Structured vortex light with orbital angular momentum (OAM) holds promise for various optical applications, but its on-chip electrical detection remains challenging. Here, the authors report the realization of photothermoelectric OAM detectors based on 2D PdSe_2_ and spin-Hall surface plasmonic polariton couplers.

## Introduction

Suppling a new degree of freedom for light, the optical orbital angular momentum (OAM) in vortex beams has drawn extensive attention owing to its tremendous application potential in optical manipulation^1–3^, imaging^4^, and communications^5,6^. Particularly, the virtue of theoretically unlimited orthogonal OAM states can provide more channels to boost the information capacity and enhance security in optical communications^7,8^. The detection of vortex structured light is a fundamental process in classical and quantum optical communications. The optical OAM detections recently developed based on diffraction and interference methods using such as log-polar coordinate transformation^9^ and optical gratings^10,11^ require bulky and complex setups, thereby imposing a fundamental limit towards achieving on-chip photonic systems. Therefore, on-chip OAM detectors with filter-less configurations and direct electric readout, are highly desired at the micro- and nano-scale to match the size of optical devices for the next-generation compact high-capacity nanophotonic applications.

Extensive theoretical and experimental methods towards achieving on-chip OAM detection have been explored, such as surface plasmon polariton (SPP)-based OAM detection^12,13^ and orbital photogalvanic effect (OPGE)-based OAM photodetector^14^. The SPP-based OAM detection method can separate various OAM modes into different spatial positions using focused SPPs, which are excited by the vortex beams with well-designed plasmonic nanostructures, such as nanoring slit^15,16^ and nanograting^17,18^. For example, the nonresonant mode-sorting sensitivity enabled on-chip parallel multiplexing over a bandwidth of 150 nm in the visible wavelength range is realized^12^. However, these SPP-based detection methods require far-field detectors to characterize the transferred SPPs or near-field scanning microscope to record the focused SPPs, thus limiting their applications in system-level integrations that require on-chip OAM electrical detection. On the other hand, to directly measure the OAM–related SPP, integrating holographic based plasmonic interfaces with a commercial silicon photodiode can provide a feasible strategy to directly detect the OAM eigenstate of a vortex beam^13^. However, the holographic coupler designed based on a typical OAM mode cannot be used to effectively distinguish other OAM modes. Moreover, to achieve electric readout of OAM modes, the OPGE has been firstly proposed to directly detect OAM modes by using a 2D Weyl semimetal material^14^. However, this method requires extracting the OPGE current by simultaneously measuring the left- and the right-circular polarization dependent photocurrents, which limits the detection accuracy and the detection speed. Up to now, on-chip OAM photodetectors with electrical readout has not been demonstrated experimentally.

Here, to realize all-on-chip OAM detection, we design an on-chip OAM detector based on a 2D photothermoelectric (PTE) material, namely palladium selenide (PdSe_2_), integrating with a spin-Hall SPP coupler, which can directly translate the angular momentum (AM) to photoelectric outputs. Different from the previous SPP-based methods, the spatially sorted SPPs modulated by both spin angular momentum (SAM) and OAM are directly translated into the corresponding amplitude and polarity of PTE responses in PdSe_2_ nanoflake, which possess broadband absorption and giant thermoelectric effect. On the other hand, our on-chip OAM detectors with a filler-less structure show a good detection stability and accuracy comparing with the OPGE-based OAM photodetectors. Experimental demonstrations in the long-wave infrared region show that the PTE responses are strongly dependent on the OAM of incident vortex light with a fixed SAM. In addition, the chirality together with the ellipticity of the scalar vortex beam with a fixed OAM mode can also be detected by selecting proper port-pairs. Furthermore, the on-chip vortex light photodetector with both SAM and OAM sensitivity realized in the long-wave infrared region could promote the development of laser-based ground-ground free space communications. Our study provides a promising route to develop all-on-chip photodetector for vortex structured light detections, thus accelerating implementations OAM-based optical imaging, communications, and quantum information processing.

## Results

### Design of the on-chip photodetectors for vortex light

**Fig. 1:**
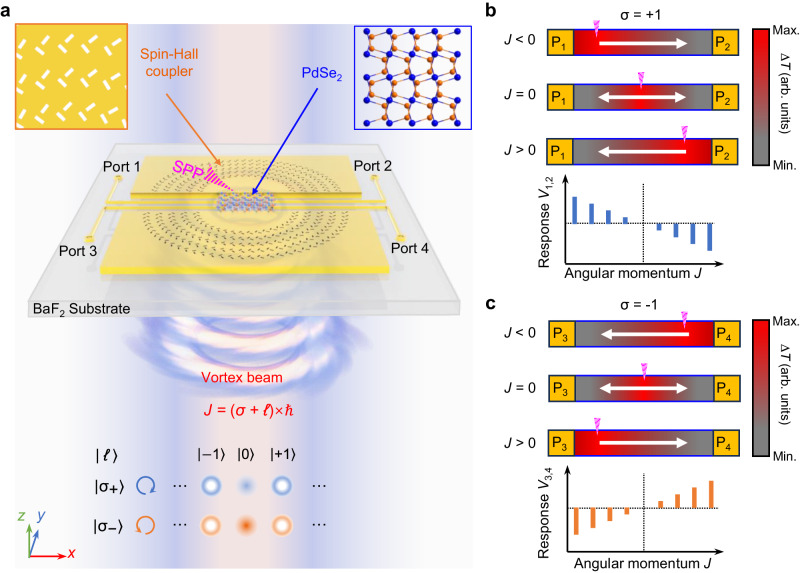
Design principles of our on-chip photodetector for vortex light. **a** Schematic of the on-chip photodetection of vortex beams with total optical angular momentum *J* = (σ + *ℓ*) ℏ, where σ is the modal index for spin angular momentum, *ℓ* is the topological charge for orbital angular momentum, and ℏ is Planck’s constant divided by 2π. The designed photodetector consists of a metallic spin-Hall surface plasmonic polariton (SPP) coupler and a thermoelectric detector based on two-dimensional materials PdSe_2_ with four ports. The angular momentum dependent responses were measured by recording the photovoltages in an open circuit. **b**, **c** Schematic of the temperature distribution introduced by SPP excitation converted from vortex beams (top panel) with different orbital angular momentums and angular momentum dependent responses (bottom panel) for spin angular momentum σ = +1 (**b**) and σ = −1(**c**). ∆*T*: temperature change. White arrows indicate the major carrier diffusion directions. Pink triangles indicate the focusing SPP positions.

The optical angular momentum (AM) includes the spin angular momentum (SAM) which corresponds to the polarization singularity with helical electric vector and the orbital angular momentum (OAM) which corresponds to the phase singularity with helical wavefront. It can be evaluated by *J* = (*σ* + ℓ) ℏ, where *σ* is the modal index of the SAM, *ℓ* is the topological charge for the OAM, and ℏ is Planck’s constant divided by 2π^19^. Figure 1a illustrates the concept of our on-chip AM detection for vortex structured light. Here, a set of AM-carrying monochromatic vortex beams of σ = ±1, and *ℓ* = 0, ±1, ±2…are adopted as the excitation sources. Two semi-ring-shaped spin-Hall plasmonic couplers with well-designed nanostructures can separate various AM modes by spatially focusing the surface plasmonic polaritons (SPPs) which are launched by the vortex excitation sources based on constructive interferences^15,18,20^. Particularly, the top (bottom) semi-ring coupler is used for sorting the left- and right-handed circularly polarized vortex beams with σ = +1 and σ = −1, respectively. Subsequently, the spatially focused SPP hot spots are absorbed by the two-dimensional thermoelectric materials introducing a corresponding spatial temperature gradient along the *X*-direction. The two-dimensional thermoelectric material chosen in this study is an exfoliated palladium selenide (PdSe_2_) nanoflake, which possesses a high Seebeck coefficient, a broadband light absorption property, and a good air-stability^21,22^. Finally, the photovoltage responses arising from the photothermoelectric (PTE) effect, can be obtained from the corresponding port-pairs, which are strongly dependent on the total AM *J*. As shown in Fig. 1 b, for the σ_+_ vortex beams, the positions of focused SPP hot spots spatially shift from the left to the right when angular momentum *J* changes from a negative to a positive value. As a result, the corresponding photovoltage responses *V*_12_ of port-pair P_1,2_ of port 1 (P_1_) and port 2 (P_2_) can directly represent the AM states in both amplitudes and polarities. Based on the same operation principle, the photovoltage responses *V*_34_ can directly reflect the AM states of σ_-_ vortex beams (Fig. 1c).

In terms of the spin-Hall plasmonic coupler design, two hierarchical structures are considered to realize simultaneous detection of both polarization and phase singularities (Supplementary Note 1). The primary nanostructure is designed to be a chiral plasmonic coupler consisting of two parallel columns of apertures in a metal film, which can unidirectionally launch SPP wave under the illumination of circularly polarized light^20^. The secondary structure is designed to be a semi-ring-shaped grating which is used to focus the as-launched SPP waves into spatially separated hot spots for various OAM-carrying vortex beams^15^. Integrating these two hierarchical structures allows us to simultaneously detect AM-superposed vortex beams.

### Sorting angular momentum states with the surface plasmons

In principle, the sorting of angular momentum states can be realized in any frequency regime. As a proof of concept, we design the spin-Hall coupler operating in the long-wave infrared regime to verify our design concepts. Firstly, we employ numerical full-wave simulations using the finite-difference time-domain (FDTD) method to optimize the single aperture with a linear polarized light excitation. The single aperture designed for operation at λ = 8 μm with an optimized width *W* = 0.7 μm and length *L* = 2.8 μm can launch a SPP wave with a wavelength of λ_SPP_ ≈ 8 μm at the gold-air interface. In addition, the chiral plasmonic coupler made up of five column pairs can launch a unidirectional SPP wave under the illumination of circularly polarized light (Supplementary Fig. 3). The directionality of SPP propagation can reach up to 0.98, which is defined as *D*_SPP_ = (*I*_L_ − *I*_R_)/(*I*_L_ + *I*_R_), where *I*_L/R_ is the SPP intensity towards the left- and the right-side of the coupler, respectively. This ultrahigh directionality of as-designed coupler ensures the capacity of separating the vortex beams with different SAM modes. Furthermore, the primary chiral plasmonic coupler is bent into a ring and shows focusing and de-focusing capabilities for the right- and the left-circularly polarized lights, respectively (Supplementary Fig. 4). Secondly, we fabricate a chiral plasmonic coupler made up of 10 parallel column pairs in a 200 nm-thick gold film on a barium fluoride (BaF_2_) substrate with the focused ion beam milling (Methods). The optimized geometrical parameters of the structure are *S* = 2 μm and *D* = 4 μm. By measuring both the transmission and the reflection, we can get a peak coupling efficiency of as-fabricated coupler around 5.6% at 8 μm (Supplementary Fig. 5).

**Fig. 2:**
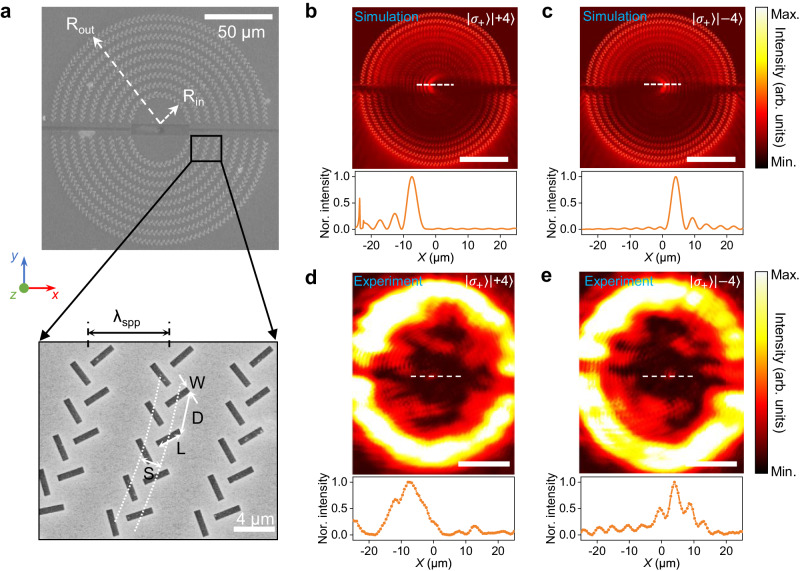
Spatial separability of angular momentum states with spin-Hall coupler. **a** Scanning electron microscope (SEM) image of a fabricated ring-shaped spin-Hall coupler for operation at 8 μm. The inside and outside radius are 21 μm and 91 μm, respectively. Multiple concentric column pairs made of rectangular apertures with structure parameters of *W* = 0.7 μm, *L* = 2.8 μm, *D* = 4 μm, and *S* = 2 μm, are spaced λ_SPP_ = 8 μm apart. **b**, **c** Numerical simulations of electric-field intensity distributions (top panel) and corresponding line cut profiles (bottom panel) of the normalized intensity in the spin-Hall coupler excited by σ_+_ vortex beams with topological charge *ℓ*  =   + 4 (**b**) and *ℓ*  =   − 4 (**c**). **d**, **e** Experimental measured intensity distributions (top panel) and corresponding line cut profiles (bottom panel) of the normalized intensity in the spin-Hall coupler excited by σ_+_ vortex beams with topological charge *ℓ*  =   + 4 (**d**) and *ℓ*  =   − 4 (**e**). Scale bars in **b**–**e**: 50 μm.

To confirm the predictions of the angular momentum sorting, we fabricate the as-designed spin-Hall plasmonic coupler consisting of two hierarchical structures, as shown in Fig. 2a. The coupler is consisted of two semi-ring shaped multiple concentric column pairs, which are spatially separated by 10 μm in *Y*-direction. The inside and outside radii of the semi-ring coupler are set to be 21 and 91 μm, respectively, to match the sizes of the generated vortex beams with various topological charges in experiments. Moreover, the as-generated vortex beams are calibrated using the interferometric method and characterized (Supplementary Note 2 for details). The recorded interference patterns for vortex beams with topological charges ranging from −4 to 4, show a fork-like shape. The number of branches and the orientation of the fork fringes reveal the absolute value and sign of the topological charge accordingly^23^. The circular polarization of the generated vortex beam with a topological charge of *ℓ* = +1 is characterized by using a polarizer. The same donut shapes recorded with different polarizer angles indicate a good circular polarization state of the as-generated vortex beam.

Based on the experimental setups, numerical simulations are performed to study the spatial separability of angular momentum states with the spin-Hall coupler. As shown in Fig. 2b and c, under the excitation by σ_+_ vortex beams, the SPP waves are focused by the top semi-ring coupler, and the focused SPP hot spots are spatially shifted to the left and the right for vortex beams with topological charge *ℓ*  =   + 4 and *ℓ*  =   − 4, respectively. In addition, the SPP focal position (*X*_j_) along *X*-direction is linearly dependent on the total optical angular momentum modal index *j* = σ + *ℓ*, which can be expressed by: *X*_j_ = *k* × *j*, where *k* denotes the distance between any two neighboring SPP focal position^15^. The SPP focal positions for σ = +1 and σ = −1 vortex beams are physically separated by 10 μm in the *Y*-direction based on the selection of the chiral plasmonic coupler (Supplementary Fig. 11). The distance *k* between any two neighboring OAM modes is about ±1.5 μm and the spot size is about 5 μm, indicating a distinguishable separation for different AM modes (Supplementary Fig. 12). The distance-voltage resolution is influenced by many factors such as the device channel scale, the active materials, and the photoresponse mechanism. Further analyses have revealed that the distance between the focused spots is 120 nm and 310 nm for the incident wavelength of 633 nm and 1550 nm, respectively^15^. Such sub micrometer-scale distances of adjacent SPP focal spots in the visible and near-IR region are still possible to obtain a stable SNR readout by using a small device channel and a photovoltaic response mechanism, which has a highly local-position dependent photovoltage or photocurrent responses^24,25^. Thus, our designed structure can be extendable to the visible and near-field regimes by further optimizing the device scale and choosing working mechanism with higher local-position dependent photoresponse, such as photovoltaic response. Furthermore, to experimentally verify the sorting capacity of as-fabricated spin-Hall plasmonic coupler, far-field imaging detection is employed with a microscopy set-up (Supplementary Fig. 6)^26^. The measured field distributions are in good agreement with the numerical simulations (Fig. 2d, e). Distinctive spatial shift of SPP hot spot can be observed for σ_+_ vortex beams with topological charge *ℓ*  =   + 4 and *ℓ*  =   − 4. The spatial shift of the focused SPP hot spots is also observed for σ_-_ vortex beams with topological charge *ℓ*  =   + 3 and *ℓ*  =   − 3 (Supplementary Fig. 13). The annular intensity distribution with high brightness around the center corresponds to the scattered light of the directly transmitted light through the couplers. Note that the sizes of the as-measured SPP hot spots are slightly bigger than that of the simulated near-field values because of the divergence of the output SPP propagating towards the far-field. On the other hand, the vortex beams with different OAM modes are generated using the spiral phase plate with finite thickness-levels, which also make the experimental results slightly different from the simulation results.

### Thermoelectric detection of the AM-superposed vortex light

**Fig. 3:**
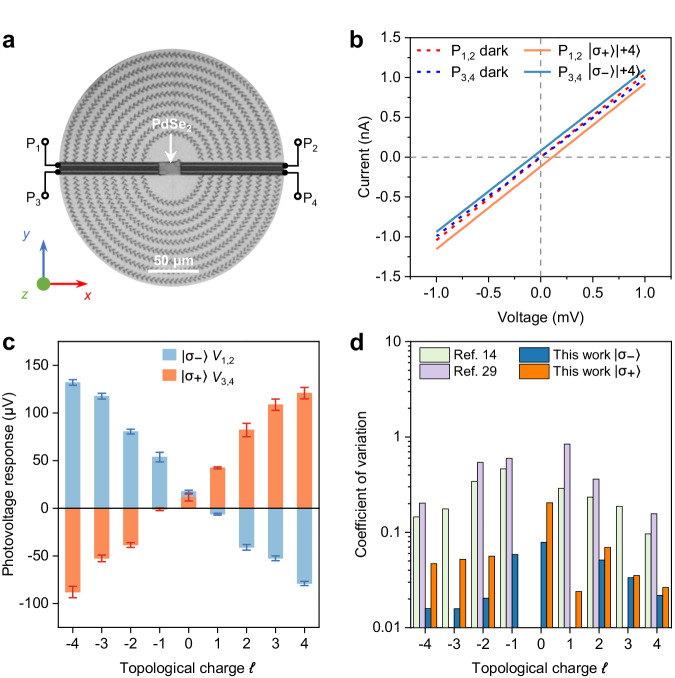
Photovoltage response to the angular momentum states with high reliability. **a** Optical image of a fabricated on-chip photodetector. **b** *I*-*V* curves in dark, σ_+_, and σ_-_ vortex lights illumination conditions with topological charge *ℓ* = +4 for ports P_12_ and P_34_, respectively. **c** Experimentally measured photovoltage responses for σ_+_ and σ_-_ vortex beams with topological charge ranging from −4 to 4. The error bars are the standard deviation of five measurements. **d** Comparison of the coefficient of variation of angular momentum detection.

To obtain photoelectrical detection of AM modes, the photothermoelectric effect is introduced to directly readout the spatially discriminated SPP focusing hot spots owing to its strong position-dependent photoresponses. As shown in Fig. 3a, the exfoliated PdSe_2_ nanoflake is used as the active photothermoelectric material, and four bottom-contact electrodes are used as four output ports (P_1_ to P_4_). Four output ports are separated by 10 μm and 8 μm along the *X*- and the *Y*-direction, respectively, to match the distributions of SPP focusing hot spots for AM modes of σ = ±1 and *ℓ*  =  0, ±1, ±2, ±3, ±4 as shown in Supplementary Fig. 12d. Both the two-dimensional spatial separability of as-designed coupler and the strong position-dependent PTE response provide the physical grounds for the AM-carrying vortex light detection. Firstly, as shown in Fig. 2b, the *I*-*V* curves for two port-pairs (P_1,2_ and P_3,4_) measured in the dark condition show linear behaviors indicating a good ohmic contact between the electrodes and the PdSe_2_ nanoflake, which is important for the PTE responses^27,28^. Then, the *I*-*V* curves for corresponding port-pairs under the illumination of vortex beam at a wavelength of 8.0 μm and a topological charge of *ℓ*  = +4 are measured (Fig. 2b). Both curves are parallel to those measured under dark conditions and show obvious shifts towards to the up and the down directions of the P_1,2_ and the P_3,4_, respectively, thereby indicating a negative and positive zero-biased photothermoelectric responses accordingly. Although the PTE responses are sensitive to the light intensity gradient, its effect can be excluded because the hollow area with a diameter over 100 μm (Supplementary Fig. 7) of the donut-shape intensity of the as-generated vortex beam is significantly larger than the size (10×15 μm^2^) of the active material, which means the incident vortex beam cannot directly interact with the active material. Notably, although the light intensity at center is not zero for the incident beam with OAM mode of *ℓ*  = 0, the effect of the intrinsic light intensity gradient on the PTE response is negligible because of the symmetric electrode’s configuration and symmetric light intensity distribution. Therefore, we can confirm that the observed PTE responses are excited by the focal SPP hot spots, and these opposite polarity of photovoltage responses are arising from the opposite linear dependence of SPP focal position on the topological charge for the top and the bottom couplers (Supplementary Fig. 12).

As discussed above, the proposed on-chip photodetector is properly designed to be able to directly readout the AM modes of the incident vortex lights. To demonstrate the discrete behavior of the PTE responses, a set of vortex beams with AM modes of σ = ±1 and *ℓ*  =  0, ±1, ±2, ±3, ±4 is generated sequentially based on a Laguerre-Gaussian (LG) beam using a series spiral phase plates and a quarter-wave plate. The photovoltage responses (*V*_1,2_ and *V*_3,4_) from the port-pairs P_1,2_ and P_3,4_ for σ_-_ and σ_+_ vortex beams, respectively, are measured with a constant optical power (125 mW) and the topological charge *ℓ* ranging from -4 to 4 (Supplementary Fig. 14). As shown in Fig. 3c, the experimentally measured photovoltage responses clearly show a high correlation with the topological charge *ℓ* for both SAM mode σ = +1 and σ = −1. Aside from the intensity of the focal SPP hot spots, the PTE response in our case is also dominated by the position of the focal SPP hot spots. The photovoltage responses are almost linearly dependent on the topological charge *ℓ* for a fixed SAM mode, which is consistent with the relationship between the SPP focal position and the topological charge *ℓ*. Therefore, we can conclude that the measured photovoltage responses are mainly dominated by the positions of the focal SPP hot spots, which forms the central basis of our angular momentum detector. In addition, the photovoltage responsivity of the detector for the σ_+_ vortex beam with topological charge *ℓ* = +4 is about 1.0 mV W^−1^ obtained by fitting the photovoltage response as a function of the incident laser power ranging from 10 mV to 125 mV (Supplementary Fig. 15). Meanwhile, the photovoltage (photocurrent) responsivity of the detector can be calculated to be *j* × ±0.2 mV W^−1^ (*j* × ±0.2 nA W^−1^), which depends on the total optical angular momentum. The fast response speed of the detector is also investigated with a rise/decay time of 69/25 μs, indicating a low reading duration for the AM modes detection (Supplementary Fig. 16).

To quantify the photoresponse with respect to the integer angular momentum modes, we use the coefficient of variation of the measured photovoltage response for each angular momentum mode to evaluate the reliability of the angular momentum photodetector. As shown in Fig. 3d, the calculated coefficient of variation for our proposed detector is in the range of 0.01-0.08, which is an order of magnitude smaller than that of the previous detectors based on the OPGE^14,29^. The lower coefficient of variation of our detector is owing to the steady state of the directly electric readout, rather than the dynamic extraction of the OPGE current. Compared with other recent angular momentum detectors, on top of a higher reliability, our proposed detector also exhibits a lower reading duration, a longer operation wavelength, and can detect more angular momentum combinations. However, comparing with other OAM photodetection methods, the responsivity of our detector has no superiority because of the relatively low coupling efficiency of the coupler and the relatively high propagation loss of SPP (Supplementary Table 1)^13,14,29^.

### Polarization states detection of the scalar vortex beams

**Fig. 4:**
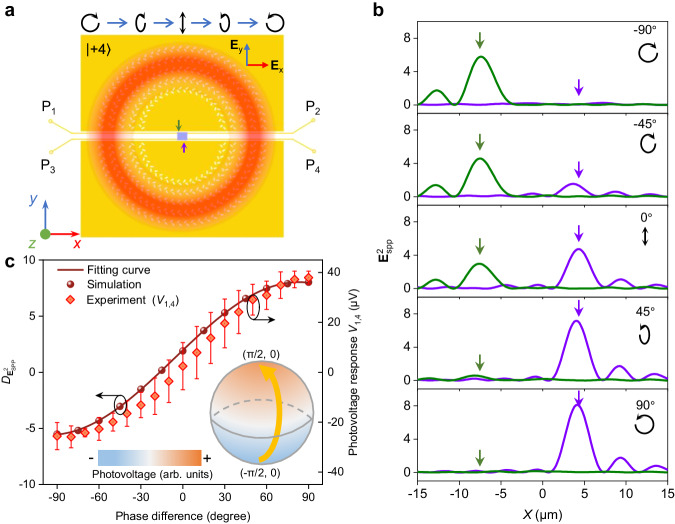
Polarization states dependent thermoelectric responses of the scaler vortex beams. **a** Schematic of the polarization states detection of the vortex beams with a fixed topological charge *ℓ* = +4. **E**_x_ and **E**_y_ denote the components of electrical field for the x and y axes. Green and purple arrows indicate two typical positions of SPP focusing spots. **b** Line cuts of SPP intensity profiles along the top (green line) and bottom (purple line) edge of the PdSe_2_ flakes excited by vortex beams with phase difference between **E**_x_ and **E**_y_ changing from −90° to 90°. **c** Simulated SPP intensity difference (${D}_{{{{{{\rm{E}}}}}}_{{{{{\rm{SPP}}}}}}^{2}}$) between two focusing positions and Experimental measured photovoltage responses as a function of phase differences between **E**_x_ and **E**_y_ for vortex beams with topological charge *ℓ* = +4. The inset shows a phase-difference-dependent and azimuthal-angle-independent distribution of photovoltage response *V*_1,4_. The error bars are the standard deviation of four measurements.

Polarization is also one of the important features of light, which characterizes the electric field oscillation and is essential for various applications such as optical communication, remote sensing, and navigation^30–32^. As demonstrated above, two optical SAM eigenstates including left- (σ_+_) and right-handed (σ_-_) circular polarization states can be selected by the spin-Hall plasmonic coupler successfully. Furthermore, here we study the detection capability of our detector for more general polarization states of the scalar vortex beams, where the polarization states are space-invariant. As displayed in Fig. 4a, our experimental configuration for the polarization state detection is also the four-ports device but use the port-pairs P_1,4_ to record the PTE response outputs, and the incident light is set to be a LG vortex beam with a fixed OAM mode (*ℓ* = +4) but various polarization states. By changing the phase difference between two components of electrical field for the x (**E**_x_) and y (**E**_y_) axes from −90° to 90°, a series of polarization states changing from LCP to RCP through linear polarization can be obtained, as indicated by the black arrows in Fig. 4a. Firstly, we perform numerical simulations to study the SPP intensity distributions under the illumination of different polarized vortex beams (Supplementary Fig. 17). With the phase difference changes from -90° to 90°, the focal SPP intensities localized at two typical positions as indicated by green and purple arrows change gradually with the opposite trends (Fig. 4b).

To further reveal the relationship between the focal SPP intensities in two typical positions and the polarization states, we calculate the SPP intensity difference by: ${D}_{{{{{{\rm{E}}}}}}_{{{{{\rm{SPP}}}}}}^{2}}=\,{E}_{{{{{{\rm{SPP}}}}}},{{{{{\rm{L}}}}}}}^{2}-{E}_{{{{{{\rm{SPP}}}}}},{{{{{\rm{R}}}}}}}^{2}\propto \sin \delta$, where, ${E}_{{{{{{\rm{SPP}}}}}},{{{{{\rm{L}}}}}}}^{2}$ and ${E}_{{{{{{\rm{SPP}}}}}},{{{{{\rm{R}}}}}}}^{2}$ denote the SPP intensity at left and right positions, $\delta$ denote the phase difference between E_x_ and E_y_, which is related to the PTE responses directly (Supplementary Note 3). As shown in Fig. 4c, the intensity difference as a function of phase difference is fitted well with a sine function, which is consistent with the previous results^20^. In our experiments, the photovoltage responses for different polarization states are recorded by continuously rotating a QWP angle with a fixed topological charge *ℓ* = +4 (Supplementary Fig. 18). In a period of 180°, the polarization states of light changes in the order of, e.g., linear at 0° – right-handed circular at 45° – linear at 90° – left-handed circular at 135° – linear at 180°. Moreover, the photovoltage responses with changing the phase difference are plotted in Fig. 4c. The experimentally measured photovoltage responses agree well with the simulation results. Here, the photovoltage response *V*_1,4_ can reflect both the sign and value of phase difference, which corresponds to the chirality and ellipticity of the polarization states, respectively. Notably, as the designed spin-Hall SPP coupler is independent on the azimuthal angle, we cannot distinguish the linear polarization states with different azimuthal angles (see the inset of Fig. 4c). Thus, we can claim that the chirality and ellipticity of the scalar vortex beam can be effectively detected using a properly ports pair in our on-chip detector.

## Discussion

In summary, we have demonstrated that by means of angular momentum mode sorting capability of the spin-Hall SPP coupler, the phase information of the vortex beam can be translated to the amplitude and sign of the photovoltage responses. The on-chip photodetection of the angular momentum modes of vortex lights will facilitate the high-capacity optical communications and quantum information processing based on angular momentum division^33,34^. Notably, the performance of the as-proposed detector can be further boosted through improving the coupling efficiency with phase-gradient metasurface^35,36^ and reduce the propagation loss of surface plasmonic polariton^16,37^. On the other hand, the response sensitivity of the as-proposed detector should be further enhanced by improving the heat-to-electricity conversion efficiency and the figure of merit of the thermoelectric materials^38^. In addition, the demonstrated detection mechanism could be extended to other optical detectors in other wavelength regimes with properly designed working mechanism, device configuration, and well-designed plasmonic couplers^39^. Moreover, the on-chip angular momentum converting strategy may open up unexplored opportunities for improving the detectivity in other wave fields such as electron and acoustic waves.

## Methods

### Device fabrication

As the first step to device fabrication, four electrodes and two semicircles are patterned on the double-polished barium fluoride (BaF_2_) substrate using the standard electron-beam lithography (EBL) method. Then, a 5-nm-thick Cr and 200-nm-thick gold film are deposited by electron-beam evaporation and followed by lift-off process. To improve the quality of the gold film, the as-prepared sample is thermally annealed under a temperature of 700 °C for 1 hour. Thereafter, the surface plasmonic polariton couplers are fabricated using the focus ion beam (FIB) milling method with an accelerating voltage of 30 kV and a 28-pA beam current. The PdSe_2_ nanoflake mechanically exfoliated from a bulk crystal with suitable lateral scales is finally selected and put on the specific position of the chip with electrodes by a dry-transfer method.

### Optical and electrical measurements

The optical spectra are obtained using a Fourier Transform Infrared spectrometer (FTIR, Bruker) with a microscope (Thermo Fisher). The circularly polarized light is generated by combining a linear polarizer and a quarter-wave plate. For the OAM beam characterization, an IR beam profiler (DATARAY, S-WCD-IR-BB-30) is used to record the intensity distribution.

The angular momentum dependent photoresponse is measured by using a homemade photoresponse measurement system where the infrared light with different polarization states and orbital angular momentum modes is obtained from a quantum cascade laser (Daylight Solutions, MIRcat) with tunable wavelengths in the range of 7–11 μm combining a series of half-wave plate (Edmund, #85-122), quarter-wave plate (Edmund, #85-115), and spiral phase plate (HOLO/OR, VL-253-8000-Y-A), and then focused on the backside of samples using a zinc selenide IR focusing objective (Innovation Photonics, LFO-5-12). The generated photovoltages from four ports are then recorded in sequence by a highly sensitive source-measure unit (Keysight, B2912A). The voltage noise is measured by using a lock-in amplifier (Zurich Instruments, HF2LI).

### Numerical simulation methods

The full-wave simulation of the surface plasmon polariton of the spin-Hall grating is done using Lumerical FDTD Solutions. The simulated structure consists of a BaF_2_ substrate, gold film (200 nm thickness) with spin-Hall gratings and air. The single subwavelength aperture is optimized by recoding its absorption to plane wave with a circular polarization. The radius interval of semi-ring gratings is equal to the SPPs wavelength (λ_SPP_ ≈ 8 μm) at the Au-air interface. A doughnut-shaped intensity distribution, a helical wavefront, and the circular polarization are adopted in the setup of the incident beam.

### Supplementary information

Supplementary Information

### Source data

Source Data

Transparent Peer Review file

## Data Availability

The Source Data underlying the figures of this study are available with the paper. All raw data generated during the current study are available from the corresponding authors upon request. Source data are provided with this paper.
