# Supplementary material for: On-chip photodetection of angular momentums of vortex structured light
Source: Nat Commun. 2024 Jun 26;15:5396. doi: 10.1038/s41467-024-49855-0 (PMC11208514; doi:10.1038/s41467-024-49855-0)
Supplement: Supplementary file 1 — Supplementary Information [file 41467_2024_49855_MOESM1_ESM.pdf]

Supplementary Information

for

On-chip photodetection of angular momentums of vortex structured light

Mingjin Dai[1], Chongwu Wang[1], Fangyuan Sun[1], and Qi Jie Wang[1,2]

[1]School of Electrical and Electronic Engineering, Nanyang Technological University, Singapore 639798, Singapore.

[2]Centre for Disruptive Photonic Technologies, School of Physical and Mathematical Sciences, Nanyang Technological University, Singapore 637371, Singapore.

Corresponding Author: qjwang@ntu.edu.sg

Table of contents:

Supplementary Notes 1-3

Supplementary Figures 1-18

Supplementary Table 1

Supplementary References 1-6

Supplementary Note 1: Design of the spin-Hall plasmonic coupler

The spin-Hall plasmonic coupler is designed based on two hierarchical structures as shown in Supplementary Figure 1. Particularly, the primary structure is a chiral plasmonic couplers made of two column apertures according to previous work[1]. Firstly, we consider the surface plasmonic polariton (SPP) launched at the air-gold interface by a single aperture. The SPP wave vector can be calculated by:

$$k_{SPP} = k_0\sqrt{\frac{\varepsilon_{air}\varepsilon_{Au}}{\varepsilon_{air}+\varepsilon_{Au}}} \tag{S1}$$

where, k_0 is the wave vector of free space incident light, ε_{air} and ε_{Au} are dielectric constants of air and metal Au. The wavelength of SPP is calculated by $\lambda_{SPP} = \frac{2\pi}{k_{SPP}}$ for the operation wavelength of 8 μm. As shown in Supplementary Figure 2, with well-designed geometric structure parameters, the single aperture launches the SPP with a wavelength $\lambda_{SPP} \cong 8\ \mu\text{m}$, which agrees well with the theory. On the other hand, the single aperture shows a resonance extinction peak at λ = 8 μm.

Secondly, the apertures are arranged into two columns with appropriate geometric structure parameters as shown in Supplementary Figure 1. When the lateral spacing S of the columns is set to be $S = \frac{\pi}{2}k_{SPP}^{-1}$, the SPP field intensities propagating to the right (I_R) and the left (I_L) to the pair of columns can be calculated by:

$$I_L \propto [(E_1^2 + E_2^2) - 2E_1E_2\sin\delta] \tag{S2}$$

$$I_R \propto [(E_1^2 + E_2^2) + 2E_1E_2\sin\delta] \tag{S3}$$

where, E_1 and E_2 denote the amplitudes of the field components coupled to the respective columns and δ denotes the relative phase between the E_1 and E_2. As a result, the chiral plasmonic coupler made up of five column pairs can launch a unidirectional SPP wave under the illumination of circularly polarized light, as shown in Supplementary Figures 3 and 4.

Thirdly, when the chiral plasmonic couplers are bent into a semi-ring shape, the SPP can be tracked based on the Huygens–Fresnel principle[2]. As shown in

Supplementary Figure 1c, considering that the incident light has an electric field of $E = e^{i(\sigma+\ell)\phi}(\vec{e}_r + i\sigma\vec{e}_\phi)$ with a circularly polarization state, the electric field at the surface is given by:

$$U(\vec{r}) = \int_0^\pi \exp(ij\phi) \frac{\exp(ik_{SPP}|\vec{R}-\vec{r}|)}{\sqrt{|\vec{R}-\vec{r}|}} d\phi \tag{S4}$$

where, $j = \sigma + \ell$ is the total optical angular momentum modal index. By employing the paraxial approximation, the position at the peak focal point can be analytically expressed by:

$$x_j = sgn(j)\frac{\chi_j}{k_{SPP}} \tag{S5}$$

where, χ_j is the first non-null zero point of the derivative of electric field distribution along x-direction. This means that the focal positions are totally determined by the optical angular momentum modal index j and the wavelength λ_{SPP} of SPPs.

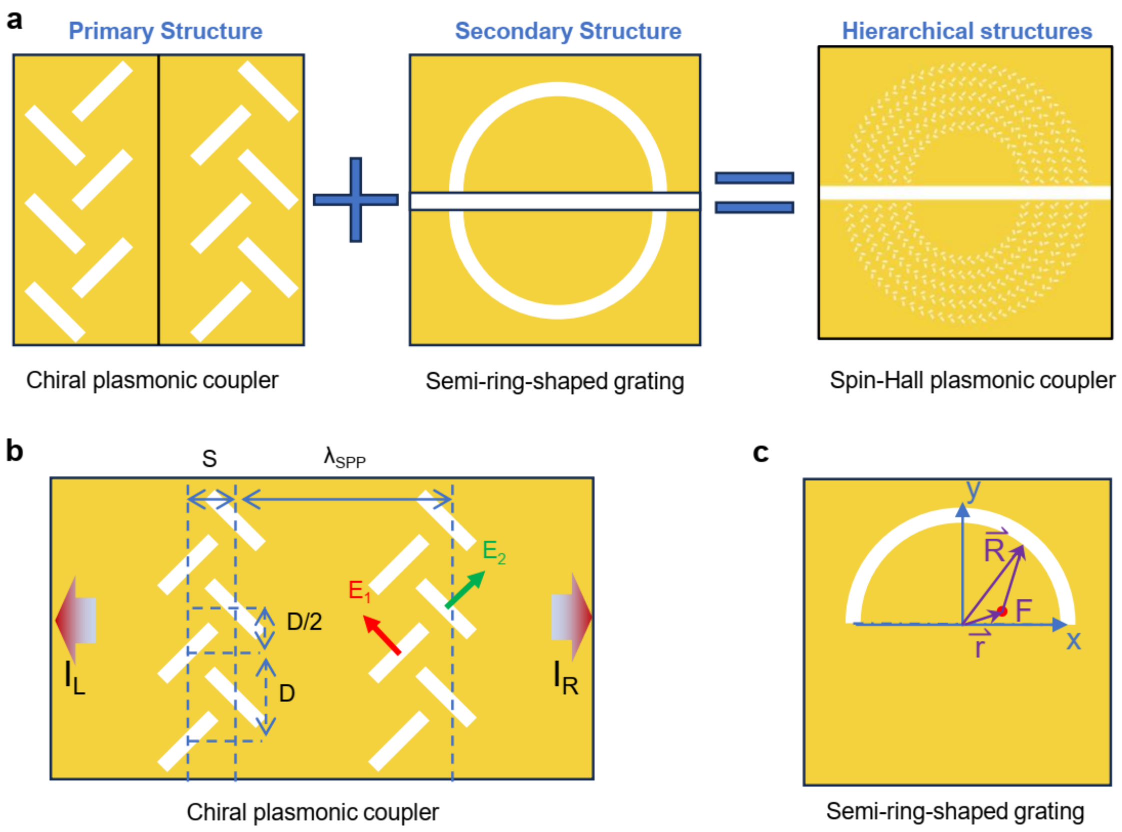

Supplementary Figure 1 | Design principle of the spin-Hall plasmonic coupler for sorting various angular momentum states. a, The primary chiral plasmonic couplers are bent into semi-ring shape to form the final hierarchical structures, which is used for sorting various superposed angular-momentum states including the spin and the orbital angular momentums. **b**, The chiral plasmonic coupler is made of column pairs spaced by λ_{SPP}. Two columns of apertures are positioned in parallel with spacing S. The columns couple to the field components $\mathbf{E}_1$ and $\mathbf{E}_2$ of the incident field **E**. The resulting SPP plane waves propagate to the right and the left side away from the column pair with intensities I_R and I_L. **c**, Mechanism of the sorting of the semi-ring-shaped grating. The $\vec{R}$ is the position vector of any point on a semi-ring slit, $\vec{r}$ denotes the position vector of any viewpoint F.

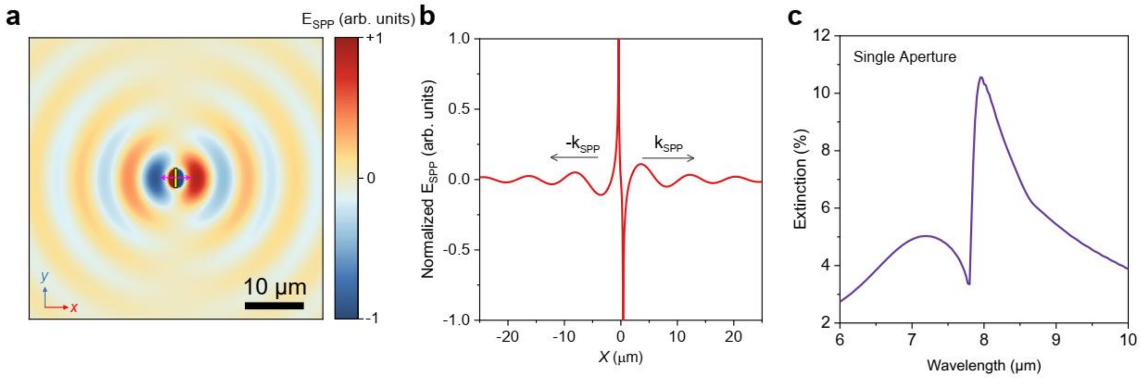

Supplementary Figure 2 | Simulation of surface plasmonic polariton launched by a single aperture. a, The calculated normal component of the SPP electric field launched by a subwavelength rectangle aperture by scattering incident light polarized perpendicular to its long axis. The pink arrow indicates the polarization of the incident light. **b**, Line cut of SPP electric field profile shows the launched surface plasmonic polariton propagates along the *x* axes. k_{SPP} denotes the SPP wave vectors. **c**, The simulated extinction spectrum of as-designed single aperture shows a peak at 8 μm.

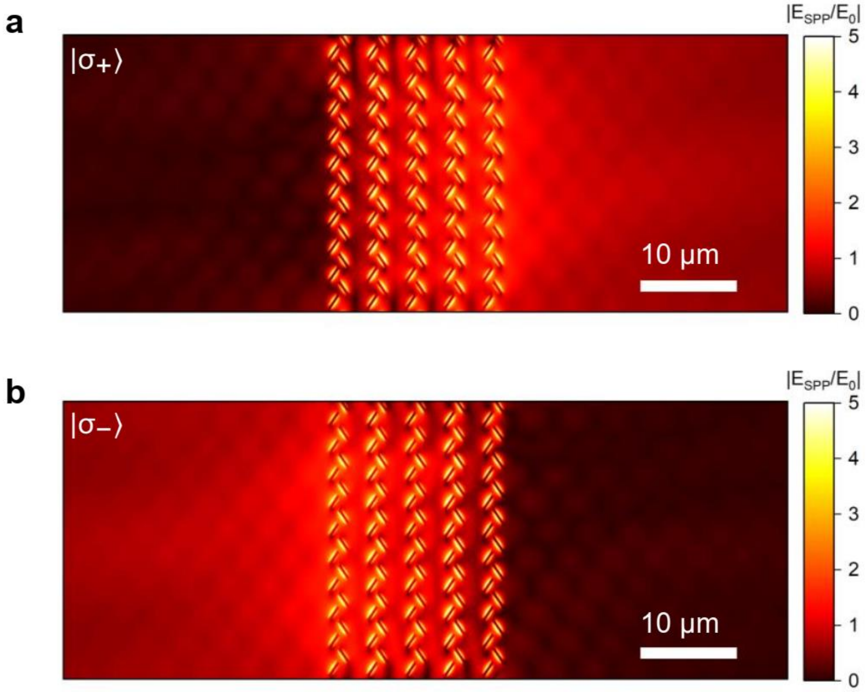

Supplementary Figure 3 | Simulation of surface plasmonic polariton launched by multiple column pairs. a,**b**, Simulated electric field of SPP (normalized by electric field of the incident light) above a coupler made up of five column pairs under illumination by the incident light with the left σ_+ (**a**) and the right σ_- (**b**) circular polarization states.

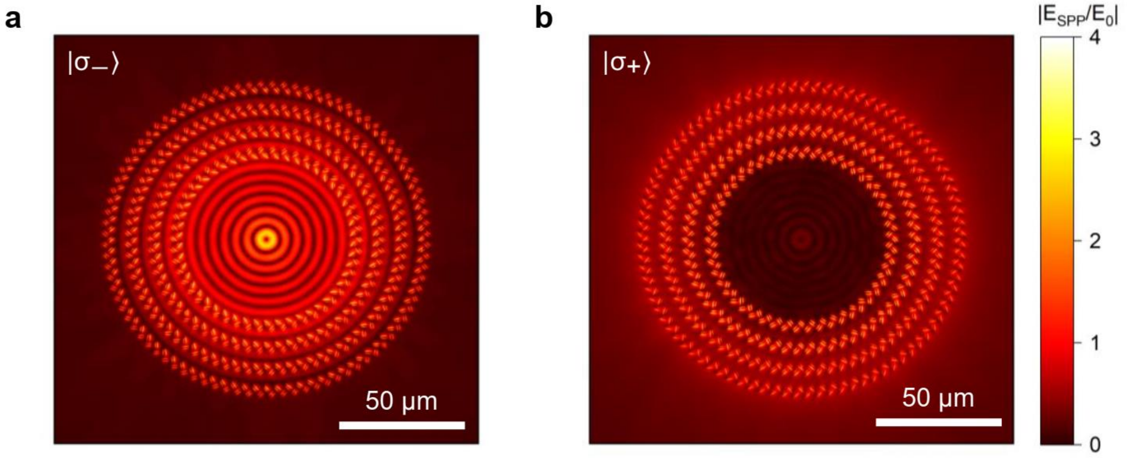

Supplementary Figure 4 | Simulation of surface plasmonic polariton launched by multiple concentrically ringed column pairs. **a**,**b**, Simulated electric field of SPP (normalized by electric field of the incident light) above a coupler made up of four concentrically ringed column pairs under illumination by a plane wave with the right σ_- (**a**) and the left σ_+ (**b**) circular polarization states.

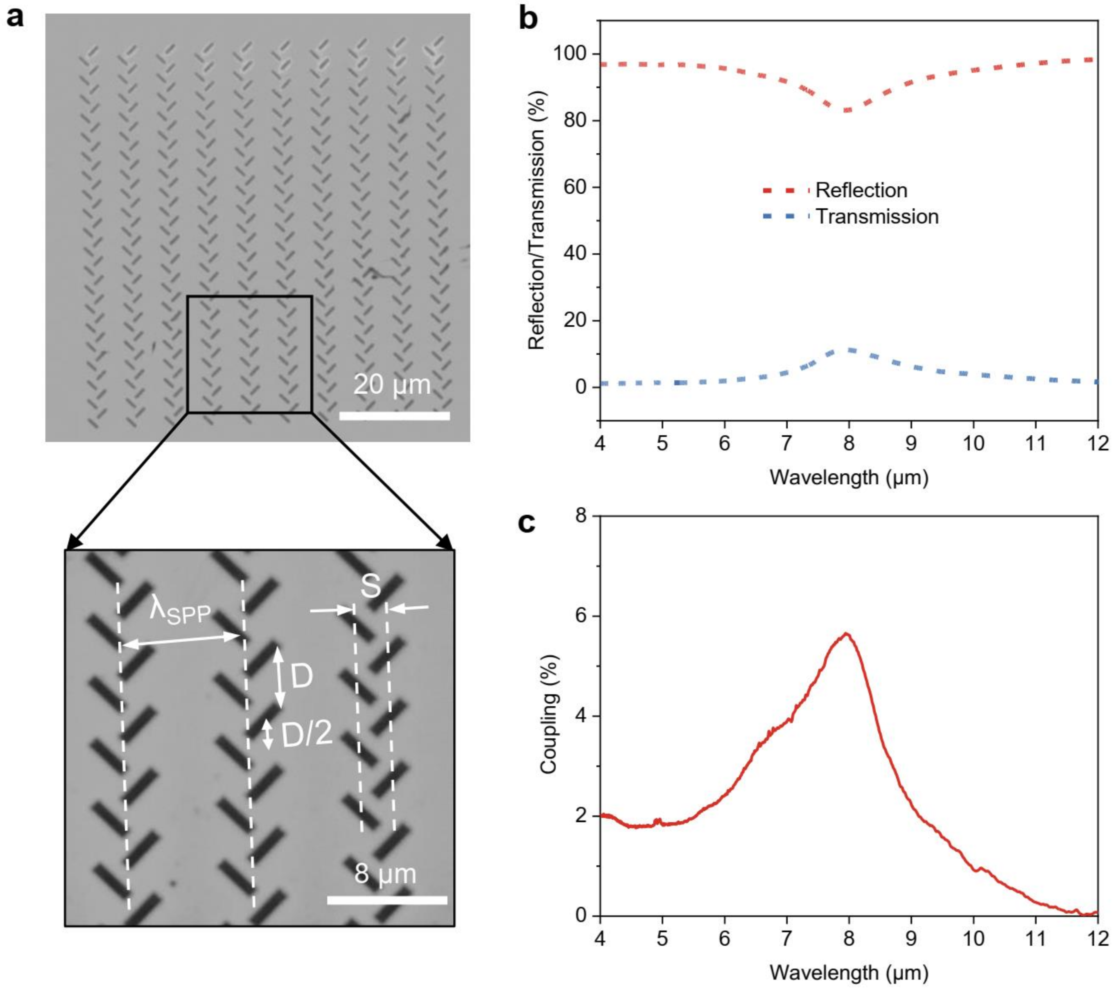

Supplementary Figure 5 | Coupling efficiency of the SPP coupler. a, Optical image of fabricated SPP coupler made up of 10 column pairs with a size of 80×80 μm^2. The structure parameters S and D denote the distances between apertures in one column along X- and Y-directions, respectively. **b**, Measured reflection (R) and transmission (T) spectra of the as-fabricated SPP couplers showing a resonance peak located at around 8 μm. c, The coupling efficiency (C) of SPP coupler calculated by using C=1-R-T, shows a maximal coupling efficiency of 5.6% at 8 μm.

Supplementary Note 2: Generation, characterization, and calibration of the vortex beams

Generation: The setup to generate series vortex beams with different combinations of the spin and the orbital angular momentum (SAM and OAM) modes is shown in Supplementary Figure 6. Here, starting from a Laguerre-Gaussian (LG) beam generated by a commercial quantum cascade laser (QCL), the LG beam passes through half-wave plate (HWP), quarter-wave plate (QWP), and spiral phase plate (SPP), and becomes a vortex beam with typical SAM and OAM modes. Both HWP and QWP are used to control the polarization state to realize circularly polarized beam corresponding to the SAM mode. The SPP with different topological charges are used to control the phase distribution of as-generated beam corresponding to the OAM mode.

Characterization: After generating the vortex beams, the size and polarization are characterized using the setup as shown in Supplementary Figure 6. Firstly, the intensity profile of the vortex beams with topological charge of 0, +1, +2, +3, and +4 are recorded by an infrared beam profiler. As shown in Supplementary Figure 7, all the intensity distributions show a donut shape except the beam with a topological charge of zero. The diameters of corresponding generated vortex beams are 54, 132, 150, 177, and 186 μm, respectively. Secondly, a polarizer is set in the front of beam profiler to characterize the circular polarization state of the as-generated vortex beam. As shown in Supplementary Figure 8, the intensity distributions of the generated vortex beams with different polarizer angles show same donut shape, indicating a good circular polarization state.

Calibration: More importantly, the phase distributions of as-generated vortex beams are calibrated by using the interferometric method[3]. Supplementary Figure 9 shows the corresponding setup. One Laguerre-Gaussian beam (“1”) and one vortex beam (“2”) with typical spin and orbital angular momentums intervene at BS-2 and the fork pattern is then recorded by the beam profiler. The recorded interference patterns for vortex beams with topological charges ranging from -4 to 4, show a fork-like shape. The number of branches and the orientation of the fork

fringes reveal the absolute value and sign of the topological charge accordingly (Supplementary Figure 10).

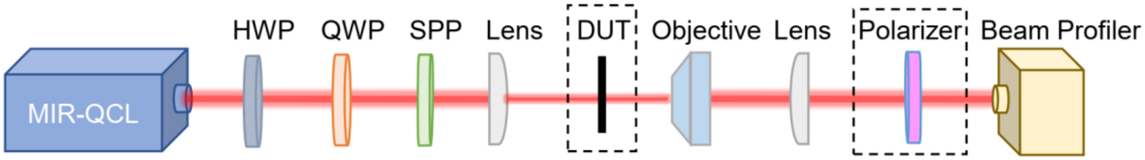

Supplementary Figure 6 | Setup for generation, characterization, and detection of vortex beams with angular momentums. HWP and QWP denote half-wave plate and quarter-wave plate, respectively. SPP denotes the spiral phase plate. DUT denotes the device under test. Dashed boxes denote the removable part for vortex beam characterization and detection.

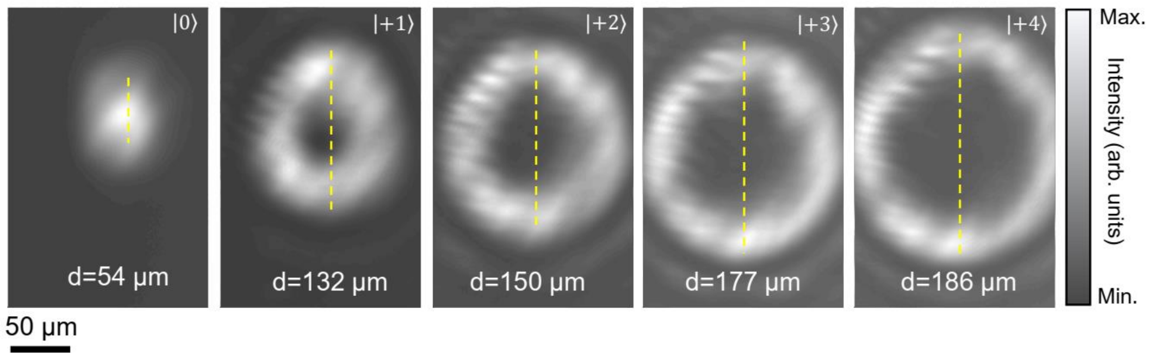

Supplementary Figure 7 | Sizes of vortex beams with topological charges ℓ ranging from 0 to +4. The intensity profile of the vortex beams with topological charge of 0, +1, +2, +3, and +4 as recorded by an infrared beam profiler. The diameters of corresponding generated vortex beams are 54, 132, 150, 177, and 186 μm, respectively.

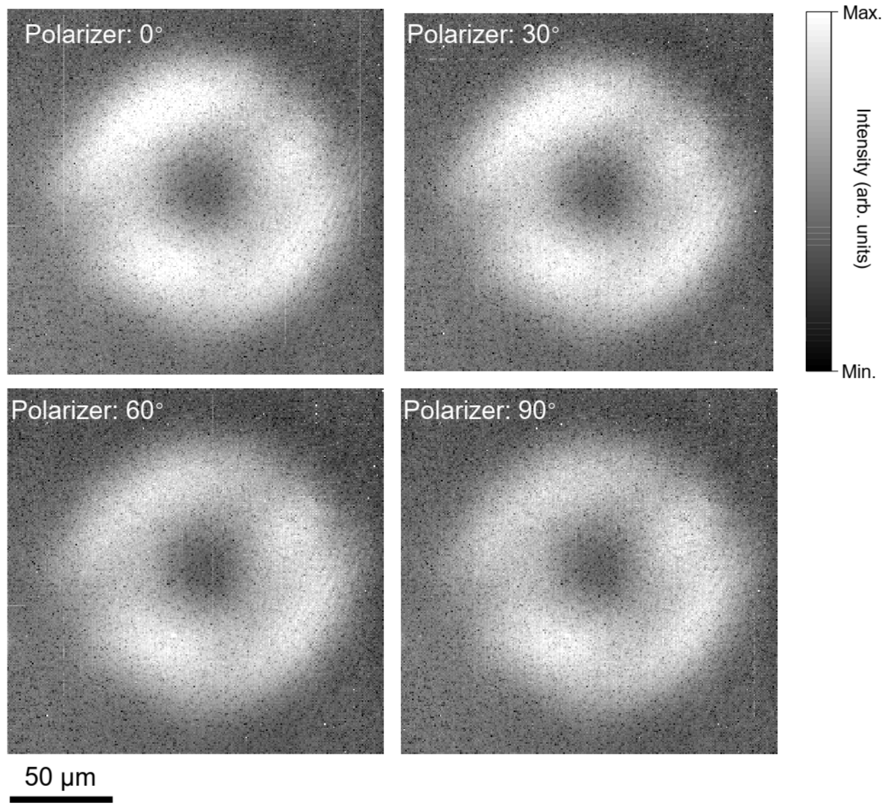

Supplementary Figure 8 | Characterization of the circularly polarized vortex beams with topological charge ℓ = +1. The intensity distributions of the generated vortex beams with different polarizer angles show same donut shape indicating a good circular polarization state.

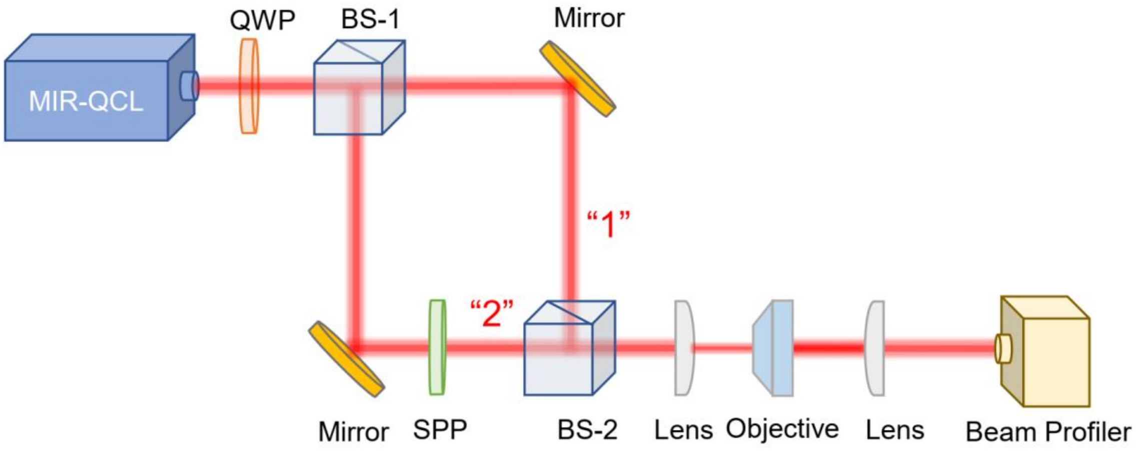

Supplementary Figure 9 | Setup for calibration of vortex beams with angular momentum. QWP and SPP denote quarter-wave plate and spiral-phase plate, respectively. BS denotes the beam splitter. One gaussian beam ("1") and one vortex beam ("2") with typical spin and orbital angular momentums intervene at BS-2 and the fork pattern is then recorded by the beam profiler.

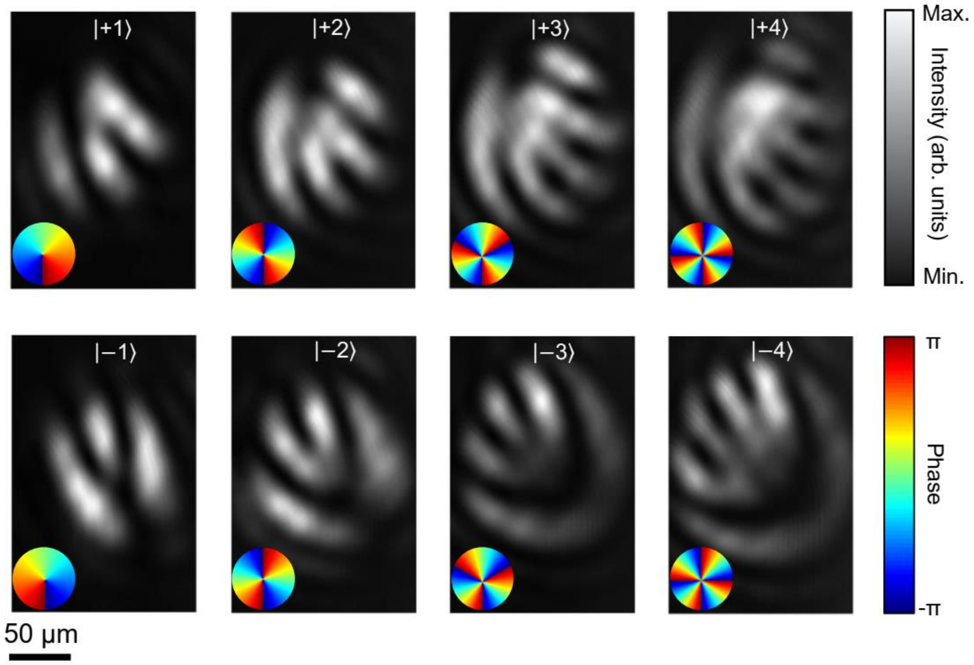

Supplementary Figure 10 | Calibration of the vortex beams with topological charge ℓ ranging from -4 to +4. The recorded fork patterns for vortex beams show a topological charge dependence. Insets are the phase distribution of corresponding vortex beams.

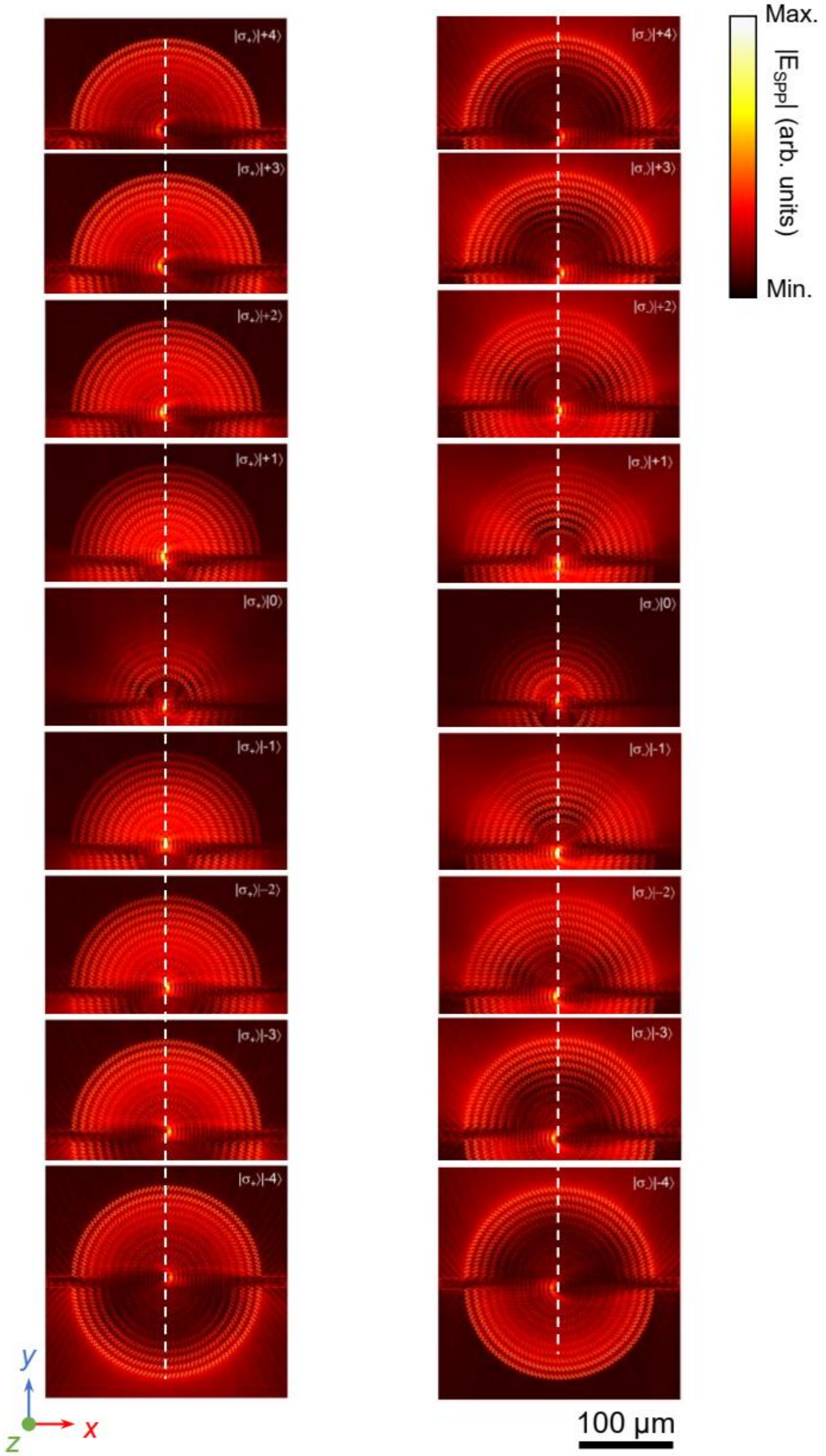

Supplementary Figure 11 | Numerical simulation of the SPP intensity distribution excited by σ_- and σ_+ vortex beams with topological charge ℓ ranging from -4 to +4. The white dashed line indicates the origin position of x axes.

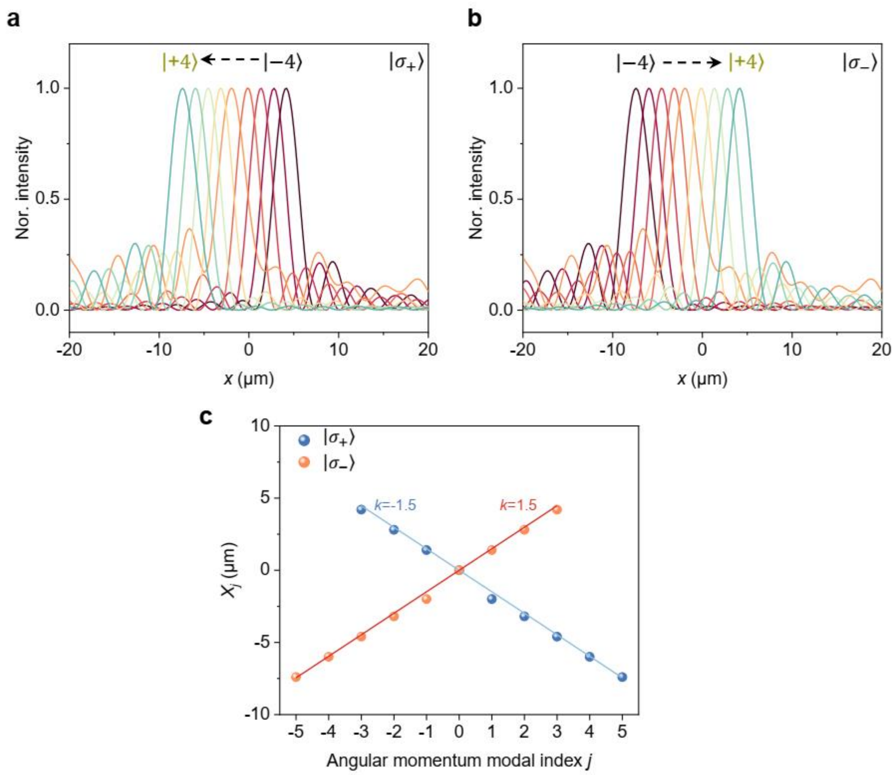

Supplementary Figure 12 | Numerically simulated line cuts of the intensity profiles of SPP launched by σ_- and σ_+ vortex beams with topological charge ranging from -4 to +4. a,**b**, Normalized line cut of SPP intensity profile excited by σ_- (**a**) and σ_+ (**b**) vortex beams with different topological charges. The distance between any two neighboring vortex beam modes is about 1.5 μm and the lateral (along x) spot size is about 5 μm. **c**, The linear fitting of the SPP focal position as a function of total optical angular momentum modal index *j* shows a distance *k* between any two neighboring vortex beam modes is about 1.5 μm. **d**, Two-dimensional position distribution of the SPP focal hot spots for different vortex beams and the four-ports device configuration.

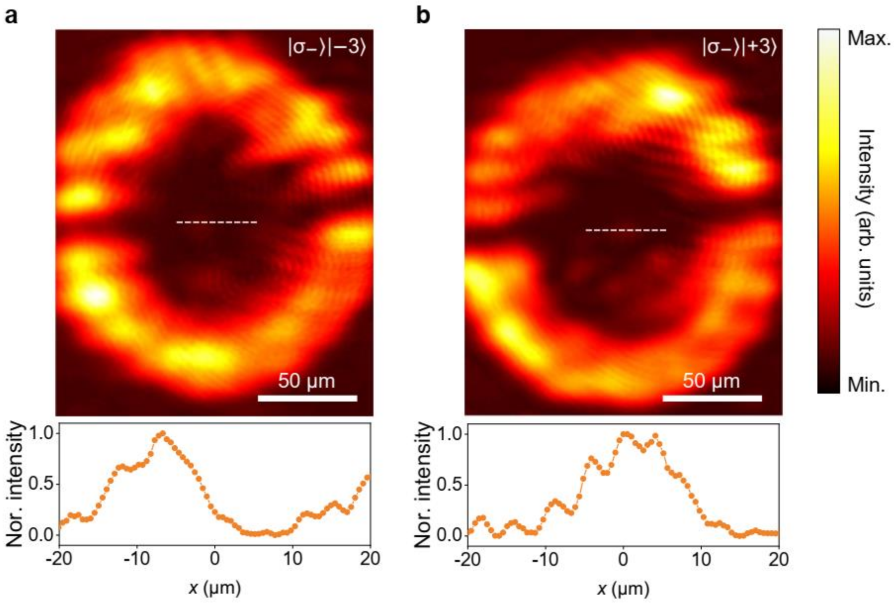

Supplementary Figure 13 | Experimentally measured intensity profiles of SPP launched by σ_- and σ_+ vortex beams with topological charge ℓ = -3 and ℓ = +3. **a**,**b**, Experimentally measured intensity distributions (top panel) and corresponding line cut profiles (bottom panel) in the spin-Hall coupler excited by σ_- vortex beams with topological charge ℓ = -3 (**a**) and ℓ = +3 (**b**).

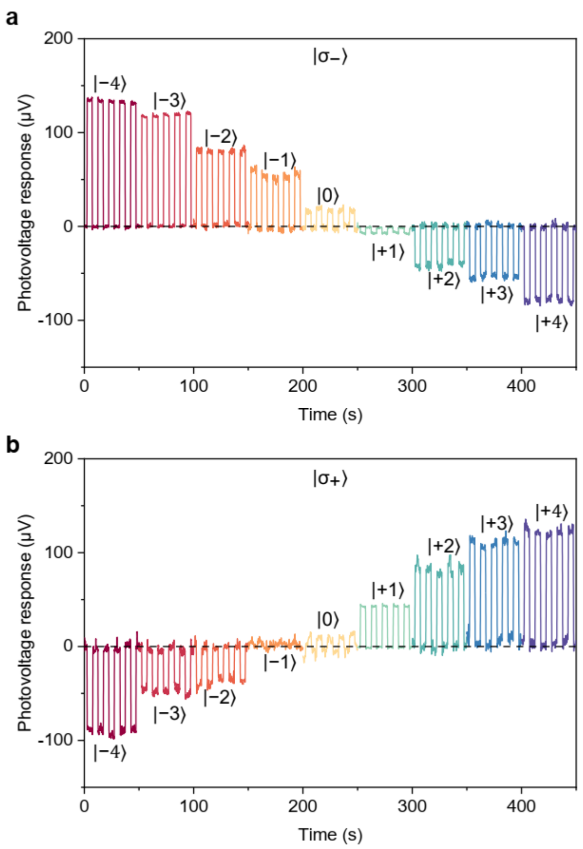

Supplementary Figure 14 | Topological charge dependent photovoltage responses. a,**b**, The temporal photovoltage responses for the σ_- (**a**) and σ_+ (**b**) vortex beams with topological charge ℓ ranging from -4 to +4.

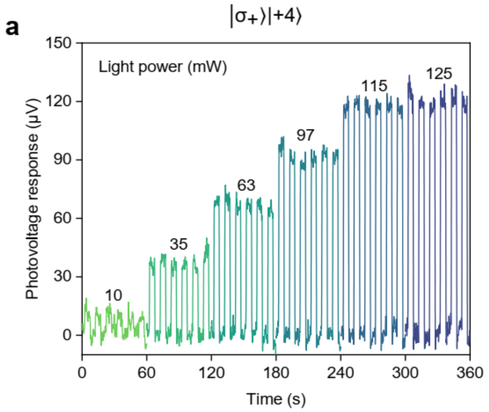

Supplementary Figure 15 | Responsivity of the detector for the σ_+ vortex beam with topological charge ℓ = +4. a, Temporal photovoltage responses with different light powers. **b**, The photovoltage response as a function of the incident light power shows a responsivity of 1.0 mV/W.

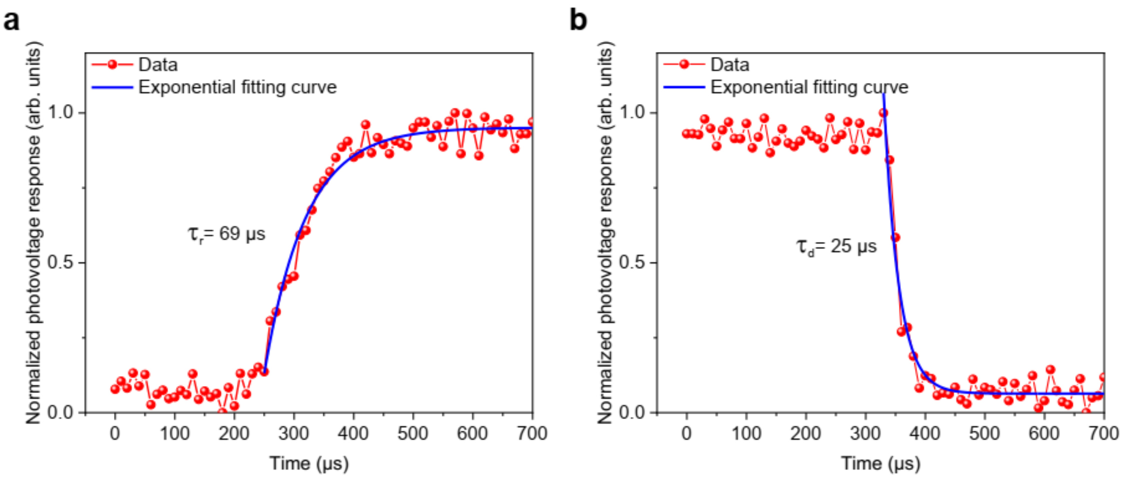

Supplementary Figure 16 | Response speed of the detector for the vortex light. a, Normalized transient photovoltage responses with a rise time constant of 69 μs. **b**, Normalized transient photovoltage responses with a decay time constant of 25 μs. Both the rise and the decay time constants are extracted by exponentially fitting as displayed in blue curves.

Supplementary Note 3: Polarization dependent SPP intensity analysis

According to equation S2 and S3, the intensity of SPP launched by the two semi-ring-shaped couplers can be as follows:

$$I_{SPP,top} \propto E_{SPP}^2 \propto \int_0^{\pi} [(E_1^2(\phi) + E_2^2(\phi)) \pm 2E_1(\phi)E_2(\phi)\sin\delta]\, d\phi \quad \text{(S6)}$$

$$I_{SPP,bottom} \propto E_{SPP}^2 \propto \int_{\pi}^{2\pi} [(E_1^2(\phi) + E_2^2(\phi)) \pm 2E_1(\phi)E_2(\phi)\sin\delta]\, d\phi \quad \text{(S7)}$$

where, $E_1(\phi)$ and $E_2(\phi)$ denote the amplitudes of the field components coupled to the respective columns and are functions of azimuthal angle ϕ.

Considering the mirror symmetry of two semi-ring-shaped couplers, the focusing SPP intensity can be further simplified as:

$$I_{SPP,top} \propto E_{SPP}^2 \propto \int_0^{\pi} [(E_1^2(\phi) + E_2^2(\phi)) - 2E_1(\phi)E_2(\phi)\sin\delta]\, d\phi \quad \text{(S8)}$$

$$I_{SPP,top} \propto E_{SPP}^2 \propto \int_0^{\pi} [(E_1^2(\phi) + E_2^2(\phi)) + 2E_1(\phi)E_2(\phi)\sin\delta]\, d\phi \quad \text{(S9)}$$

Meanwhile, the polarization states are uniform distributed for the incident scaler vortex beams, the focused SPP intensity difference between top (left) and bottom (right) coupler can be expressed:

$$D_{\mathrm{E}_{\mathrm{SPP}}^2} = E_{SPP,L}^2 - E_{SPP,R}^2 \propto \int_0^{\pi} [4E_1(\phi)E_2(\phi)\sin\delta]\, d\phi \quad \text{(S10)}$$

Furthermore, for a series polarization state with a fixed light power, the semi-circular integration of $E_1(\phi)E_2(\phi)$ is a constant term. Thereby, the focused SPP intensity difference $D_{\mathrm{E}_{\mathrm{SPP}}^2}$ is only a function of phase difference δ.

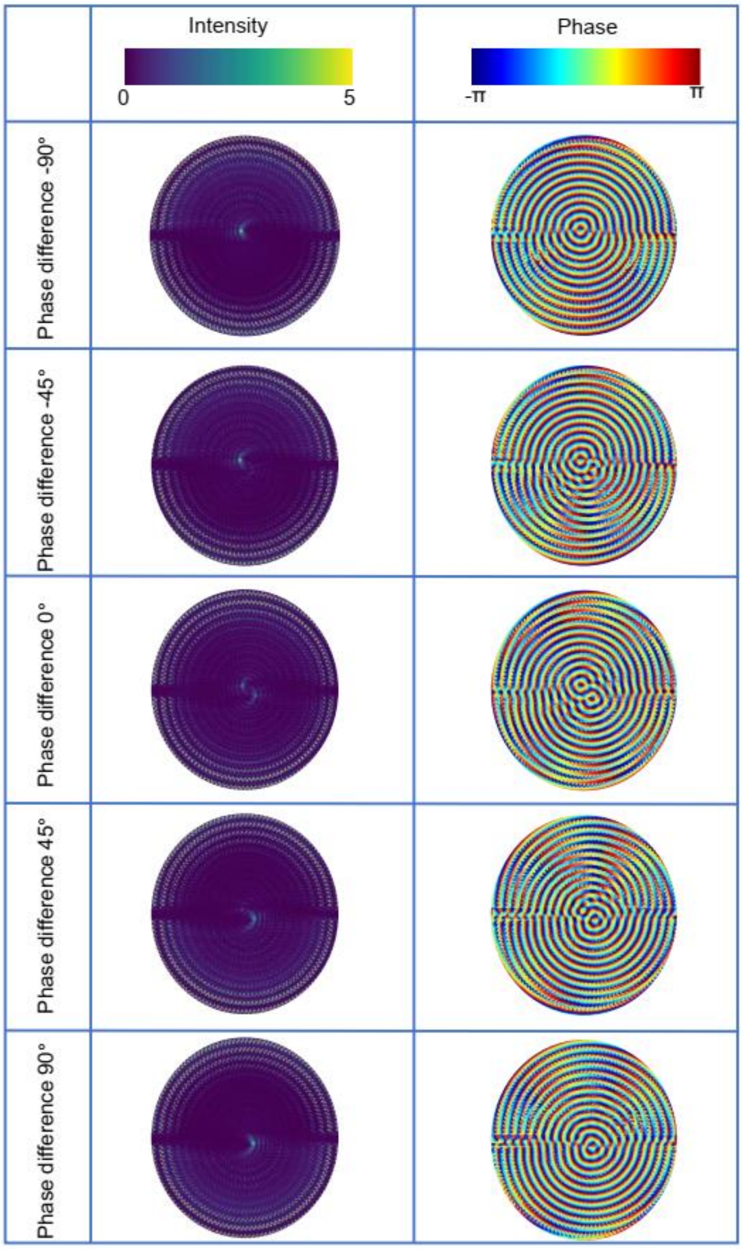

Supplementary Figure 17 | Numerical simulations of the intensity and phase distributions of the SPP excited by a set of vortex beams with different polarization states. The phase difference indicates the phase difference between components of electrical field ($\mathbf{E}_x$ and $\mathbf{E}_y$).

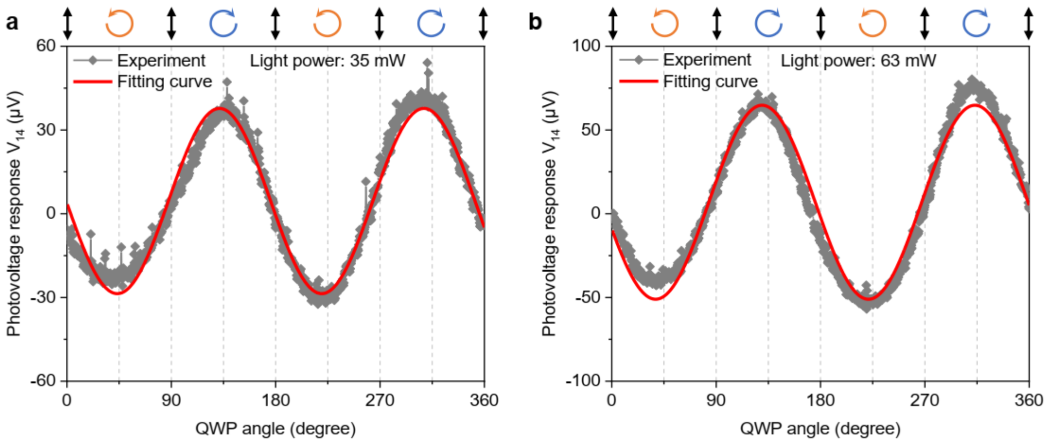

Supplementary Figure 18 | Polarization dependent photovoltage responses for vortex beam with topological charge *ℓ* = +4. a,**b**, The photovoltage responses for the vortex beams with topological charge of -4 with a light power of 35 mW (**a**) and 63 mW (**b**).

Supplementary Table 1. Performance summary of the photodetectors for orbital angular momentum detection

Materials	Mechanism	Wavelength	Reading Duration	Responsivity	OAM Modes	SAM Sensitivity	Ref.
Au/Si	Holographic SPP Coupler & Photovoltaic Effect	633 nm	N. A.	~ 1 μA/W	-1	No	4
WTe_2	Orbital Photogalvanic Effect	1 μm	N. A.	N. A.	±1, ±2, ±3, ±4	No	5
$TaIrTe_4$	Orbital Photogalvanic Effect	4 μm	25×26.5 μs	ℓ × 14.4 nA/W	±1, ±2, ±4	No	6
Au/$PdSe_2$	Spin-Hall SPP Coupler & Photothermoelectric Effect	8 μm	69 μs	j × ±0.2 mV/W (nA/W)	0, ±1, ±2, ±3, ±4	Yes	This work

SPP: surface plasmonic polariton.

ℓ: the topological charge or OAM mode number.

j: the total optical angular momentum modal index.

Supplementary References

[1] Lin, J. *et al.* Polarization-Controlled Tunable Directional Coupling of Surface Plasmon Polaritons. *Science* **340**, 331-334, (2013).

[2] Mei, S. *et al.* On-chip discrimination of orbital angular momentum of light with plasmonic nanoslits. *Nanoscale* **8**, 2227-2233, (2016).

[3] Karimi, E. *et al.* Generating optical orbital angular momentum at visible wavelengths using a plasmonic metasurface. *Light: Science & Applications* **3**, e167 (2014).

[4] Genevet, P., Lin, J., Kats, M. A. & Capasso, F. Holographic detection of the orbital angular momentum of light with plasmonic photodiodes. *Nat. Commun.* **3**, 1278, (2012).

[5] Ji, Z. *et al.* Photocurrent detection of the orbital angular momentum of light. *Science* **368**, 763-767, (2020).

[6] Lai, J. *et al.* Direct Light Orbital Angular Momentum Detection in Mid-Infrared Based on the Type-II Weyl Semimetal $TaIrTe_4$. *Adv. Mater.* **34**, 2201229, (2022).
