# Supplementary material for: On-chip photodetection of angular momentums of vortex structured light
Source: Nat Commun. 2024 Jun 26;15:5396. doi: 10.1038/s41467-024-49855-0 (PMC11208514; doi:10.1038/s41467-024-49855-0)
Supplement: Supplementary file 2 — Source Data [file 41467_2024_49855_MOESM2_ESM.pdf]

nature portfolio

Peer Review File

On-chip photodetection of angular momentums of vortex structured light

Corresponding Author: Professor Qi jie Wang

Figures originally included in the author's rebuttal have been redacted from this file.

Reviewer comments:

Reviewer #1

(Remarks to the Author)
The authors demonstrated an on-chip electrical photodetection method for structured vortex light. They 2D thermoelectric material with a spin-Hall couplers directly characterizes angular momentum modes of vortex structured light. Photothermoelectric responses in the PdSe2 nanoflake show a magnitude proportional to the total angular momentum modes of the infrared vortex beams. The electrical detection method is innovative and may find application in electronics like CMOS compatible OAM devices. Before recommending for publication, I have several questions?
1. What is the accuracy of photodetection of vortex light? Is it 100%?
2. How to explain the big differences between simulation and experimental results, like Figure2 ?
3. Compared with optical methods, what is the efficiency of this electrical detection method? Is it more efficient?
4. How to distinguish the electrical signal of intrinsic thermoelectricity and photoelectricity of the materials with the electrical signal of the OAM?
5.What are the advantages of this methods comparing dielectric metasurface detecting method?

Reviewer #2

(Remarks to the Author)
The work of Mingjin Dai et al. demonstrates an on-chip OAM photodetector based on 2D broadband thermoelectric material PdSe2. This work combines the SPP-based OAM detection method with the PTE response of the 2D thermoelectric material, and provides a direct and practical way to distinguish different OAM modes. The on-chip direct photoelectric detection of OAM is highly desired for the application of vortex light, and this work provides a promising route for developing on-chip angular momentum optoelectronic devices. Overall, I think the findings in this work is very interesting, however, I have some detailed technique concerns about the device design and the measurement. I would like to recommend it for publication providing the authors can address these questions satisfactorily.
For device design, four bottom-contact electrodes form two port-pairs to collect the PTE response for σ=±1 respectively. I'm confused about this design because the distance of focused SPP hot spots for σ=±1 in Y direction is only 5μm as calculated. Two wide electrodes seem to be enough to collect the PTE response for both σ=±1. This article lacks a theoretical explanation for this design. On the other hand, it is not clear to me how the measurement configuration can distinguish the position of the focus spot. I think the authors should provide more data and simulations to interpret, the dependence of V_ on the focal position and shows how the four electrodes design work and why it is better then two electrodes.
This article mentioned in page 10 that the PTE response is sensitive to the light intensity gradient and this effect is excluded because the ring radius of the vortex beam is much larger than the size of the thermoelectrical material. This is indeed true when the topological charge l of the vortex beam is not 0 as shown in Fig S7, but when it is 0 the intensity of the beam center is not zero and it might influence the output of the PTE response. Since the PTE responses for both |σ+⟩ and |σ-⟩ with topological charge l=0 are shown in the Fig 3c, more analysis for the impact of light intensity gradient and direct photo response from thermoelectrical materials for l=0 should be added.
The power of the OAM beam is pretty high(125mW), considering the signal noise ratio is not that good, the sensitivity is probably not high. This is fine as first demonstration of the new concept, however, I think the sensitivity of the detection should be discussed and compare reasonably with other OAM detection techniques.

Reviewer #3

(Remarks to the Author)
In the manuscript of 'On-chip photodetection of angular momentums of vortex structured light', the authors developed a vortex detector based on the spin-Hall couplers and the Photothermoelectric (PdSe2)/Au electrodes. The key contribution of this work is associated with directly electric readout, which is advantageous to the on-chip integrated systems.
Though it is an interesting work, it would be more suitable for a more specialised journal given the scope and novelty presented. Additionally, a few scientific contents and superior technological performances should be further scrutinised.

(1) The spin-orbit coupling on an interface can be flexibly modulated by designer micro-and-nano slits. It's commonly seen. Ref. 20 firstly reported the spin-dependent directional SPPs. Subsequently, quantities of papers have been studied in various applicable fields, such as plasmonic focusing (Nano Lett 15, 5739-5743 (2015)), 2D holography (Nat Commun 6, 8360 (2015)), chirality-coded devices (Nat Commun 6, 10051 (2015)). Though Ref. 15 didn't adopt a pair of nanoslits, it's conceivable that the decorated semicircular structures have the ability to discriminate the SAM and OAM. Thus, the novelty with respect to the device design is modest.

(2) The authors claim their technological superiority over the others, i.e., refs. 12 and 14. In my opinion, it should be further discussed carefully. Firstly, ref. 12 proposed a broadband metadevice, which is inaccessible to this work. Secondly, the requirement of an additional 1/4 waveplate in front of detector (ref. 14) is not intractable. A number of simple solutions can be adopted to resolve the problem, e.g., ultrathin film, miniaturized nanoantennas, etc. More importantly, the above-mentioned works implemented the SAM and OAM detection at the visible and near-IR spectral range, which is more suitable to optical communication or quantum scenarios. In contrast, the authors made the device working at the wavelength of 8 μm. To my knowledge, this spectrum suits the molecular fingerprint diagnosing, food analysis, gas sensing, etc. Would you please explain what's the specifical application that needs SAM and OAM discrimination in this spectrum?

(3) The PET material plays a vital role in the spin and orbital angular momentum mode division. Nonetheless, the discussion specific to the limitation of the distance- voltage resolution is absent. The larger topological charges of incident lights have, the higher photovoltages output ports detect. There should be a limitation with respect to the minimum distance of adjacent SPP focal spots. In your case, the distance is about 2.5 μm for λ=8 μm. For shorter wavelengths, however, such as visible and near-IR lights, the adjacent 'hot spots' could be nano scale. Is that possible to obtain a stable SNR readout? It is of great importance because this property determines the applicable expandability.

Author Rebuttal letter:

Reply to the Reviewersâ Comments

We are grateful to the constructive comments and advice provided by the reviewers on our manuscript Nature Communications (ID: NCOMMS-24-07824. Title: On-chip photodetection of angular momentums of vortex structured light). In response to these comments, we have provided necessary discussions and analyses, and revised the manuscript and the supplementary information thoroughly. We believe that we have addressed all the comments raised by the reviewers.

The corresponding amendments addressing the refereesâ concerns have been marked with yellow background for easy reference in the revised manuscript (Revised Manuscript with marks) and revised Supplementary Information (Revised Supplementary Information with marks). Our replies to the comments by the reviewers and the corresponding changes are listed below.

The following are details of our point-by-point responses to reviewersâ comments:

Reviewer #1:

Comments:

The authors demonstrated an on-chip electrical photodetection method for structured vortex light. The 2D thermoelectric material with a spin-Hall couplers directly characterizes angular momentum modes of vortex structured light. Photothermoelectric responses in the PdSe2 nanoflake show a magnitude proportional to the total angular momentum modes of the infrared vortex beams. The electrical detection method is innovative and may find application in electronics like CMOS compatible OAM devices.

Before recommending for publication, I have several questions?

Response to reviewer: We are grateful to the reviewer for the review and the positive comment of innovative electrical detection method. We also appreciate the reviewerâs constructive suggestions/comments that have greatly helped us to improve the quality and depth of our work.

Comment 1: What is the accuracy of photodetection of vortex light? Is it 100%?

Response to reviewer: We thank the reviewer for the question. We would like to discuss this question in two aspects:

On the one hand, according to the experimental results as shown in Fig. 3c, the photovoltage detection method not only can accurately detect different integral OAM modes when the fixed incident power is high enough to generate readable signals, but also can distinguish vortex beam between non-vortex beam such as a Gauss beam (topological charge â = 0) accurately. There is no overlap for photovoltage responses for neighboring vortex beams even after accounting for measurement errors under the experimental condition in the manuscript. On the other hand, apart from the vortex beam with integral OAM modes, there are some special vortex beams with fractional OAM modes (Nanophotonics 2022, 11, 241â273). However, owing to the limited sensitivity of our proposed detector in the present configuration, the proposed integrated detector may not distinguish the integral and non-integral OAM modes accurately owing to the complicated difference of SPP focal positions for different non-integral OAM modes.

Comment 2: How to explain the big differences between simulation and experimental results, like Figure2?

Response to reviewer: We thank the reviewer for raising the concern on which we didnât describe clearly. In fact, the simulation results shown in Figure 2b and 2c are the near-field distributions of the surface plasmonic polaritons excited by the OAM beams, while the experimental results shown in Figure 2d and 2e are far-field distributions of the transmitted light. Therefore, the sizes of the as-measured SPP hot spots are slightly bigger than that of the simulated values because of the divergence of the output SPP propagating towards the far-field. On the other hand, the OAM beams used in experiments are generated by using a series spiral phase plates with finite thickness-levels, which will also introduce the difference between the simulation and experimental results. Furthermore, the strong light intensity with high brightness at the ring boundary of the structure is from the scattered light of the directly transmitted light through the couplers. To clearly show the difference between the simulation and experimental results in Figure 2, we have added corresponding discussions in the Revised Manuscript, also copied below:

On page 9 of the revised Manuscript.

â¦â¦The annular intensity distribution with high brightness around the center corresponds to the scattered light of the directly transmitted light through the couplers. Note that the sizes of the as-measured SPP hot spots are slightly bigger than that of the simulated near-field values because of the divergence of the output SPP propagating towards the far-field. On the other

hand, the vortex beams with different OAM modes are generated using the spiral phase plate with finite thickness-levels, which also make the experimental results slightly different from the simulation results.

Comment 3: Compared with optical methods, what is the efficiency of this electrical detection method? Is it more efficient?

Response to reviewer: We thank the reviewer for the question. Considering the low coupling efficiency of the spin-Hall coupler in our work and the propagation loss of the surface plasmon polariton, the conversion efficiency from space to surface wave of our âelectrical detectionâ method in the current setting is not high as compared to the traditional optical methods whereby only free-space bulk optics are utilized. However, we would like to highlight that comparing with the optical OAM detection methods, our âelectrical

detectionâ method has a higher detection speed and is more advantageous for integrated chips which have higher potentials for practical applications without using bulky optics. On the other hand, the efficiency could be further improved by increasing the coupling efficiency using well-designed structure (Nat. Mater. 2012, 11, 426-431; Sci. Rep. 2017, 7, 40781.), and/or by reducing the propagation loss using suitable low-loss materials (Nat. Commun. 2018, 9, 4413.).

Comment 4: How to distinguish the electrical signal of intrinsic thermoelectricity and photoelectricity of the materials with the electrical signal of the OAM?

Response to reviewer: We thank the reviewer for raising the concern on which we didnât describe clearly. As shown in Supplementary Figure 7, the light intensity at the beam center is zero for the vortex beams with different OAM modes, which cannot introduce intrinsic thermoelectric and photoelectric responses of the materials. Notably, only for the OAM mode is 0, its intensity shape is a Gaussian distribution and will illuminate the material directly. However, there will no electrical signal owing to the symmetric device configuration and the ideal LG-Gaussian intensity distribution. We added a clear corresponding discussion in the Revised Manuscript, also copied below:

On page 10 of the revised Manuscript.

â¦â¦Notably, although the light intensity at center is not zero for the incident beam with OAM mode of ââ= 0, the effect of the intrinsic light intensity gradient on the PTE response is negligible because of the symmetric electrodeâs configuration and symmetric light intensity

distribution..â¦.

Comment 5: What are the advantages of this methods comparing dielectric metasurface detecting method?

Response to reviewer: We are thankful to the reviewer for the constructive question. Comparing with the dielectric metasurface detecting method, the advantages of the photodetection method in our work are mainly in two aspects:

1) Our approach is more suitable for on-chip photonics integration: The dielectric metasurface detecting method is a kind of free-space method that requires bulky systems comprising multiple components, which are usually not cost effective and are difficult to be implemented. While the on-chip photodetection method proposed in our work is based on two-dimensional photothermoelectric detector integrated with the spin-Hall coupler which can covert incident vortex light into surface plasmon polaritons, thus facilitating OAM beamâs applications to system-level integrations.

2) Our approach is more suitable for high-capacity optical communication: The dielectric metasurface detecting methods are usually realized by adopting interference and diffraction properties, which require the indirect counting of the stripes and lattices in the interference or diffraction patterns or recording the diffraction directions. This indirect detection method will reduce the OAM beam detection efficiency. In contrast, the on-chip photodetection method proposed in our work can electrically read out the OAM modes, which is more suitable for the application that require fast and efficient OAM detection, such as in OAM-multiplexed high-capacity optical communications.

Reviewer #2:

Comments:

The work of Mingjin Dai et al. demonstrates an on-chip OAM photodetector based on 2D broadband thermoelectric material $PdSe_2$. This work combines the SPP-based OAM detection method with the PTE response of the 2D thermoelectric material and provides a direct and practical way to distinguish different OAM modes. The on-chip direct photoelectric detection of OAM is highly desired for the application of vortex light, and this work provides a promising route for developing on-chip angular momentum optoelectronic devices. Overall, I think the findings in this work is very interesting,

however, I have some detailed technique concerns about the device design and the
4
measurement. I would like to recommend it for publication providing the authors can address these questions satisfactorily.

Response to reviewer: We are grateful to the reviewer for the review and the positive comments of this work. We also appreciate the reviewerâs constructive suggestions that helped us to improve our work.

Comment 1: For device design, four bottom-contact electrodes form two port-pairs to collect the PTE response for Ï=Â±1 respectively. Iâm confused about this design because the distance of focused SPP hot spots for Ï=Â±1 in Y direction is only 5Î¼m as calculated. Two wide electrodes seem to be enough to collect the PTE response for both Ï=Â±1. This article lacks a theoretical explanation for this design. On the other hand, it is not clear to me how the measurement configuration can distinguish the position of the focus spot. I think the authors should provide more data and simulations to interpret, the dependence of V_ on the focal position and shows how the four electrodes design work and why it is better than two electrodes.

Response to reviewer: We thank the reviewer for raising the concern on which we didnât describe clearly. Although two wide electrodes can collect the PTE response for both Ï=Â±1, the two electrodes cannot distinguish the vortex light with different Ï, or with different polarization states as shown in Figure 4. Because the positions of SPP focusing hot spots for both Ï=-1 and Ï=+1 are spatially separated in Y-direction, it needs at least two electrode-pairs (four ports) to distinguish the SAM of incident vortex light. To clearly describe this issue, we added the diagram of the SPP hot spots distribution in two dimensions in the Revised Supplementary Figure 12d. On the other hand, the PTE device showing a position-dependent response is well-known and widely proven both in our and other previous works (ACS Nano 2022, 16, 1, 295â305.; Nat. Commun. 2019, 10, 138.; Light Sci. Appl. 2020, 9, 120.). Therefore, comparing with two electrodes, the four-ports device configuration not only can distinguish both the different SAM and OAM modes simultaneously, but also can be used to detect the different polarization states for the scalar vortex beam with a fixed OAM mode. According to reviewerâs suggestion, we added a clear corresponding description in the Revised Manuscript, also copied below:

On page 9 of the revised Manuscript.

To obtain photoelectrical detection of AM modes, the photothermoelectric effect is introduced to directly readout the spatially discriminated SPP focusing hot spots owing to its strong position-dependent photoresponses. As shown in Fig. 3a, the exfoliated PdSe2 nanoflake is
5
used as the active photothermoelectric material, and four bottom-contact electrodes are used as four output ports (P1 to P4). Four output ports are separated by 10 Î¼m and 8 Î¼m along the X- and the Y-direction, respectively, to match the distributions of SPP focusing hot spots for AM modes of Ï = Â±1 and ââ=â0, Â±1, Â±2, Â±3, Â±4 as shown in Supplementary Fig. 12d. Both the two-dimensional spatial separability of as-designed coupler and the strong position-dependent PTE response provide the physical grounds for the AM-carrying vortex light detectionâ¦â¦

On page 12 of the revised Manuscript.

â¦â¦Furthermore, here we study the detection capability of our detector for more general polarization states of the scalar vortex beams, where the polarization states are space-invariant. As displayed in Fig. 4a, our experimental configuration for the polarization state detection is also the four-ports device but use the port-pairs P1,4 to record the PTE response outputs, and the incident light is set to be a LG vortex beam with a fixed OAM mode (â = +4) but various polarization statesâ¦â¦

On page 17 of the revised Supplementary Information.

REDACTED

Supplementary Figure 12 | Numerically simulated line cuts of the intensity profiles of

6
SPP launched by Ï- and Ï+ vortex beams with topological charge ranging from -4 to +4.

a,b, Normalized line cut of SPP intensity profile excited by l- (a) and l+ (b) vortex beams with different topological charges. The distance between any two neighboring vortex beam modes is about 1.5 Î¼m and the lateral (along x) spot size is about 5 Î¼m. c, The linear fitting of the SPP focal position as a function of total optical angular momentum modal index j shows a distance k between any two neighboring vortex beam modes is about 1.5 Î¼m. d, Two-dimensional position distribution of the SPP focal hot spots for different vortex beams and the four-ports device configuration.

Comment 2: This article mentioned in page 10 that the PTE response is sensitive to the light intensity gradient and this effect is excluded because the ring radius of the vortex beam is much larger than the size of the thermoelectrical material. This is indeed true when the topological charge l of the vortex beam is not 0 as shown in Fig S7, but when it is 0 the intensity of the beam center is not zero and it might influence the output of the PTE response. Since the PTE responses for both |Ï+â© and |Ï-â© with topological charge l=0 are shown in the Fig 3c, more analysis for the impact of light intensity gradient and direct photo response from thermoelectrical materials for l=0 should be added.

Response to reviewer: We thank the reviewer for pointing out the aspects which helps us to improve our manuscript. We agree with the reviewer that the intensity of the beam center is not zero for topological charge â = 0. While the effect of the intrinsic light intensity gradient on the PTE response is negligible because of the symmetric electrodeâs configuration and symmetric light intensity distribution. The PTE responses for both |Ï+â© and |Ï-â© with topological charge â = 0 shown in Fig. 3c are arising from the asymmetric SPP hot spots position as illustrated in Supplementary Figure 12. According to reviewerâs suggestion, we added a clear corresponding discussion in the Revised Manuscript, also copied below:

On page 10 of the revised Manuscript.

â¦â¦Notably, although the light intensity at center is not zero for the incident beam with OAM mode of ââ= 0, the effect of the intrinsic light intensity gradient on the PTE response is negligible because of the symmetric electrodeâs configuration and symmetric light intensity distribution..â¦.

Comment 3: The power of the OAM beam is pretty high (125mW), considering the signal noise ratio is not that good, the sensitivity is probably not high. This is fine as first demonstration of the new concept, however, I think the sensitivity of the detection should
7
be discussed and compare reasonably with other OAM detection techniques.

Response to reviewer: We thank the reviewer for the constructive comments. As the coupling efficiency of the as-designed coupler is relatively low and the propagation loss of the surface plasmon polaritons is relatively high, we agree that the responsivity of our device is not high, as well as the sensitivity. Here, we compared our detector with other OAM detection techniques on the responsivity. According to the reviewerâs suggestion, we added corresponding comparison and discussion on the detection sensitivity with other detection techniques in the Revised Manuscript, also copied below:

On page 12 of the revised Manuscript.

â¦â¦However, comparing with other OAM photodetection methods, the responsivity of our detector has no superiority because of the relatively low coupling efficiency of the coupler and the relatively high propagation loss of SPP (Supplementary Table 1)13,14,29.

Reviewer #3:

Comments:

In the manuscript of âOn-chip photodetection of angular momentums of vortex structured lightâ, the authors developed a vortex detector based on the spin-Hall couplers and the Photothermoelectric (PdSe2)/Au electrodes. The key contribution of this work is associated with directly electric readout, which is advantageous to the on-chip integrated systems. Though it is an interesting work, it would be more suitable for a more specialized journal given the scope and novelty presented. Additionally, a few scientific contents and superior

technological performances should be further scrutinized.

Response to reviewer: We are grateful to the reviewer for the review and the comments on the key contribution of this âinteresting workâ, which is advantageous to on-chip integrated systems. We also appreciate the reviewerâs constructive suggestions and comments that helped us to improve our work.

Comment 1: The spin-orbit coupling on an interface can be flexibly modulated by designer micro and-nano slits. Itâs commonly seen. Ref. 20 firstly reported the spin-dependent directional SPPs. Subsequently, quantities of papers have been studied in various applicable fields, such as plasmonic focusing (Nano Lett 15, 5739-5743 (2015)), 2D holography (Nat Commun 6, 8360 (2015)), chirality-coded devices (Nat Commun 6, 10051

(2015)). Though Ref. 15 didnât adopt a pair of nanoslits, itâs conceivable that the decorated semicircular structures have the ability to discriminate the SAM and OAM. Thus, the novelty with respect to the device design is modest.

Response to reviewer: We are grateful to the reviewer for the review and appreciate the reviewerâs constructive comments. In this work, the main novelty on the device design is to realize an on-chip photoelectric detection of vortex light with various OAM modes through the demonstration of an integrated photothermoelectric detector which can generate electrical signals to be directly read out for different OAM modes. A metallic spin-Hall coupler is adopted to convert the incident vortex beam into spatially separated focusing SPP spots. Although the spin-Hall coupler design concept was demonstrated (such as in Ref. 20 and Ref. 15), as far as we know, it has only been implemented in passive devices where optical methods were used for the detections (such as optical microscopy in the near-field and/or CCD camera in the far-field) but not in active on-chip photodetectors, mainly because it is not straightforward to realize direct detection of the generated photocurrent signals on chip in a suitable device configuration. In addition, as the reviewer kindly pointed out, even though itâs possible (exactly what we conceived and implemented it) that the decorated semicircular structures have the potential to discriminate the SAM and OAM, no other group has really tried to demonstrate the simultaneous discriminations of SAM and OAM modes even in passive structures by using our demonstrated multiple nanoslits since Ref. 15 was published in 2016. Therefore, we humbly state that our work is novel in using a dedicatedly designed device configuration which allows us to realize on-chip electrical photodetection of vortex light with various SAM and OAM modes.

Comment 2: The authors claim their technological superiority over the others, i.e., refs. 12 and 14. In my opinion, it should be further discussed carefully. Firstly, ref. 12 proposed a broadband metadevice, which is inaccessible to this work. Secondly, the requirement of an additional 1/4 waveplate in front of detector (ref. 14) is not intractable. A number of simple solutions can be adopted to resolve the problem, e.g., ultrathin film, miniaturized nanoantennas, etc. More importantly, the above-mentioned works implemented the SAM and OAM detection at the visible and near-IR spectral range, which is more suitable to optical communication or quantum scenarios. In contrast, the authors made the device working at the wavelength of 8 Î¼m. To my knowledge, this spectrum suits the molecular fingerprint diagnosing, food analysis, gas sensing, etc. Would you please explain whatâs the specifical application that needs SAM and OAM discrimination in this spectrum?

Response to reviewer: We are grateful to the reviewer for the review and appreciate the

reviewerâs constructive comments. Firstly, the comprehensive and objective discussions related to the previous works are added to the Revised Manuscript according to the reviewerâs suggestion. Although it is possible to realize the function of 1/4 waveplate by using ultrathin film or miniaturized nanoantennas, itâs still hard to realize the dynamic adjustment of chirality of the 1/4 waveplate which is required for the detection method proposed in Ref. 12 because of the fixed structures of the ultrathin film and miniaturized nanoantennas. Secondly, specific applications in the mid- and long-wave infrared are also discussed. Compared with the visible and near-IR spectral range, the long-wave infrared window (8-12ïm), which is so-called the atmosphere transparent window, shows a lower background noise interference, a higher atmospheric transmissivity, a lower atmospheric Rayleigh scattering loss (inverse proportional to the fourth power of the light wavelength Î»), and a lower atmospheric scintillation index, thus exhibiting a great potential for free-space communication such as satellite-to-satellite, satellite-to-ground, and interplanetary

communications. In addition, the demonstrated device and the concept are also applicable to other wavelength bands such as the visible and the near-infrared ranges which allow for more applications. To clearly show the research motivation and background of our work, these discussions are added in the Revised Manuscript, and copied below:

On pages 3-4 of the revised Manuscript.

â¦â¦For example, the nonresonant mode-sorting sensitivity enabled on-chip parallel multiplexing over a bandwidth of 150 nm in the visible wavelength range is realized12â¦â¦Moreover, to achieve electric readout of OAM modes, the OPGE has been firstly proposed to directly detect OAM modes by using a 2D Weyl semimetal material14. However, this method requires extracting the OPGE current by simultaneously measuring the left- and the right-circular polarization dependent photocurrents, which limits the detection accuracy and the detection speedâ¦â¦

On page 4 of the revised Manuscript.

â¦â¦Furthermore, the on-chip vortex light photodetector with both SAM and OAM sensitivity realized in the long-wave infrared region could promote the developments of satellite-to-satellite, satellite-to-ground, and interplanetary communicationsâ¦â¦

Comment 3: The PET material plays a vital role in the spin and orbital angular momentum mode division. Nonetheless, the discussion specific to the limitation of the distance-voltage resolution is absent. The larger topological charges of incident lights have, the higher photovoltages output ports detect. There should be a limitation with respect to the

minimum distance of adjacent SPP focal spots. In your case, the distance is about 2.5 Î¼m for Î»=8 Î¼m. For shorter wavelengths, however, such as visible and near-IR lights, the adjacent âhot spotsâ could be nano scale. Is that possible to obtain a stable SNR readout? It is of great importance because this property determines the applicable expandability.

Response to reviewer: We thank the reviewer for pointing out the aspects which helps us to improve our manuscript. First, the distance-voltage resolution is influenced by many factors such as the device channel scale, the active materials, and the photoresponse mechanism. The distance of adjacent SPP focal spots is dependent on the wavelength of SPP, which is also determined by the wavelength of the incident light. For example, the distance is 120 nm and 310 nm for the incident wavelength of 633 nm and 1550 nm, respectively (Nanoscale 2016, 8, 2227-2233). Such sub micrometer-scale distances of adjacent SPP focal spots in the visible and near-IR region are still possible to obtain a stable SNR readout by using a small device channel and a photovoltaic response mechanism, which has a highly local-position dependent photovoltage or photocurrent responses (Nature 2019, 570, 349â353; Nat. Nanotechnol. 2023, 18, 36â41). According to the reviewerâs suggestions, we added corresponding discussion of the limitation of the distance-voltage resolution in the Revised Manuscript, and copied below:

On page 9 of the revised Manuscript.

â¦â¦The distance-voltage resolution is influenced by many factors such as the device channel scale, the active materials, and the photoresponse mechanism. Further analyses have revealed that the distance between the focused spots is 120 nm and 310 nm for the incident wavelength of 633 nm and 1550 nm, respectively15. Such sub micrometer-scale distances of adjacent SPP focal spots in the visible and near-IR region are still possible to obtain a stable SNR readout by using a small device channel and a photovoltaic response mechanism, which has a highly local-position dependent photovoltage or photocurrent responses24,25. Thus, our designed structure can be extendable to the visible and near-field regimes by further optimizing the device scale and choosing working mechanism with higher local-position dependent photoresponse, such as photovoltaic responseâ¦â¦

On page 15 of the revised Manuscript.

â¦â¦In addition, the demonstrated detection mechanism could be extended to other optical detectors in other wavelength regimes with properly designed working mechanism, device configuration, and well-designed plasmonic couplers38â¦â¦

Reviewer comments:

Reviewer #1

(Remarks to the Author)
The authors fully address my concerns and questions. I agree with the acceptance of this paper and publication in Nature Communications.

Reviewer #2

(Remarks to the Author)
The authors have address all my concerns in the last round review, I would like to recommend it to be published in Nature Communications.

Reviewer #3

(Remarks to the Author)
I appreciate the responses prepared by the authors. The relies, however, still remain vague about three questions.
(1) The requirement of dynamic adjustment of 1/4 waveplate in ref. 14 wouldn't be a technological obstacle since liquid crystal retarders are quite accessible. And the authors mentioned that the simultaneous discriminations of SAM and OAM are unexplored by passive metadevices. Please see refs., e.g., Adv. Optical Mater. 2019, 7, 1801060, Adv. Optical Mater. 2019, 7, 1801328, Light: Science & Applications (2020) 9:95, Nanoscale, 2017,9, 4944, and Opt. Lett. 49, 198 (2024). Inspired by these works, the discrimination, even breaking the degeneracy of OAM and SAM has been feasible.
(2) Please note that the ref. 12 demonstrated a broadband device. This feature shouldn't be neglected. In addition to the working spectrum of the proposed device, the authors claim to the application of satellite-to-ground, satellite-to-satellite communications. Please double check. To my knowledge, besides radio frequency recent laser communications for satellite-to-ground often rely on the 0.8-1.55 um wavelengths. Please see Nat. Photonics 11, 502 (2017), Opt. Express 25, 28319 (2017).
(3) The robustness of PET material, i.e., photovoltaic or photocurrent resolution/limitation of detection, should be further discussed. As the authors know, the energy dissipation is very serious during the satellite-to-ground communication. Thus, the heat-to-electricity conversion efficiency, and the thermoelectric figure of merit should be complemented.

11

Author Rebuttal letter:

Reply to the Reviewersâ Comments

We are grateful to the constructive comments and advice provided by the reviewers on our manuscript Nature Communications (ID: NCOMMS-24-07824A. Title: On-chip photodetection of angular momentums of vortex structured light). In response to these comments, we have provided necessary discussions and revised the manuscript and the supplementary information. We believe that we have addressed all the comments raised by the reviewers.

The corresponding amendments addressing the refereesâ concerns have been marked with yellow background for easy reference in the revised manuscript (Revised Manuscript with marks) and revised Supplementary Information (Revised Supplementary Information with marks). Our replies to the comments by the reviewers and the corresponding changes are listed below.

The following are details of our point-by-point responses to reviewersâ comments:

Reviewer #1:

Comments:

The authors fully address my concerns and questions. I agree with the acceptance of this paper and publication in Nature Communications.

Response to reviewer: We are grateful to the reviewer for the constructive suggestions/comments that have greatly helped us to improve the quality and depth of our work.

Reviewer #2:

Comments:

The authors have address all my concerns in the last round review, I would like to recommend it to be published in Nature Communications.

Response to reviewer: We are grateful to the reviewer for the constructive suggestions/comments that have greatly helped us to improve the quality and depth of our work.

Reviewer #3:

Comments:

I appreciate the responses prepared by the authors. The relies, however, still remain vague about three questions.

Response to reviewer: We appreciate the reviewerâs constructive suggestions and comments that helped us to improve our work.

Comment 1: The requirement of dynamic adjustment of 1/4 waveplate in ref. 14 wouldnât be a technological obstacle since liquid crystal retarders are quite accessible. And the authors mentioned that the simultaneous discriminations of SAM and OAM are unexplored by passive metadevices. Please see refs., e.g., Adv. Optical Mater. 2019, 7, 1801060, Adv. Optical Mater. 2019, 7, 1801328, Light: Science & Applications (2020) 9:95, Nanoscale, 2017,9, 4944, and Opt. Lett. 49, 198 (2024). Inspired by these works, the discrimination, even breaking the degeneracy of OAM and SAM has been feasible.

Response to reviewer: We are grateful to the reviewer for the constructive comments. The main novelty of our work is to realize an on-chip photoelectric detection of vortex light with various angular momentum (AM) modes through the demonstration of an integrated active device of photothermoelectric detector which can generate electrical signals to directly read out for different AM modes.

As suggested by the reviewer, it might be a possible approach to integrate the liquid crystal retarders with the device proposed in Ref. 14, while the additional dynamic control of the liquid crystal and two electric outputs in vertical layered structures would make the approach nontrivial and introduce the complexity of the device and reduce the detection performance, which is probably the reason why it has not been tackled since the work Ref 14 was published. Here, we provide an alternative way to realize the direct on-chip photoelectrical detection of vortex light with different AM modes by using a filter-less device structure, with a good detection stability and accuracy.

On page 4 of the revised Manuscript.

â¦â¦On the other hand, our on-chip OAM detectors with a filler-less structure show a good detection stability and accuracy comparing with the OPGE-based OAM photodetectorsâ¦â¦

In addition, we agree with the reviewer that the discrimination, even breaking the degeneracy of OAM and SAM has been feasible in passive metadevices, while in the active

on-chip photodetectors, there is not any other report on the direct on-chip photoelectric detection of various AM modes by utilizing these metadevices, which we would like to humbly highlight again.

Comment 2: Please note that the ref. 12 demonstrated a broadband device. This feature shouldnât be neglected. In addition to the working spectrum of the proposed device, the authors claim to the application of satellite-to-ground, satellite-to-satellite communications.

Please double check. To my knowledge, besides radio frequency recent laser communications for satellite-to-ground often rely on the 0.8-1.55 um wavelengths. Please see Nat. Photonics 11, 502 (2017), Opt. Express 25, 28319 (2017).

Response to reviewer: We thank the reviewer for pointing out the aspects which helps us to improve our manuscript.

Firstly, based on the reviewerâs suggestion, we have mentioned the broadband detection of the device demonstrated in the visible range in Ref. 12 in the revised Manuscript. Secondly, we acknowledge that the currently developed laser free-space communication systems mainly focus on the near-infrared regime. Compared with the visible and near-IR spectral ranges, the mid/long-wave infrared window (8-12ïm) offers better potentials for laser-based free-space communication owing to its low propagation losses caused by Reilley scattering losses (Nature 556, 85â88 (2018); Laser Photonics Rev. 16, 2100414 (2022); Nat Commun 12, 3327 (2021); Nat Commun 13, 7662 (2022), which have attracted more attention recently. Therefore, the potential emerging application of the proposed device in long-wave infrared window is for the laser-based free space ground-ground communications, which can be explored to be used in earthquake relief, emergency, anti-terrorism, public security investigation and other fields (J. Phys.: Conf. Ser. 2161 012011 (2022); IEEE Communications Surveys & Tutorials, 16, 2231-2258 (2014)). As for the satellite-to-ground communication demonstration, it needs high requirements such as high-power laser source, small system volume, high-sensitive receiver, and so on (Nature 549, 43â47 (2017); Nature Photon. 11, 502â508 (2017)). We updated the corresponding discussions in the Revised Manuscript, and copied below:

On page 3 of the revised Manuscript.

â¦â¦For example, the nonresonant mode-sorting sensitivity enabled on-chip parallel multiplexing over a bandwidth of 150 nm in the visible wavelength range is realized12â¦â¦

On page 4 of the revised Manuscript.

3

â¦â¦Furthermore, the on-chip vortex light photodetector with both SAM and OAM sensitivity realized in the long-wave infrared region could promote the developments of laser-based ground-ground free space communicationsâ¦â¦

Comment 3: The robustness of PET material, i.e., photovoltaic or photocurrent resolution/limitation of detection, should be further discussed. As the authors know, the energy dissipation is very serious during the satellite-to-ground communication. Thus, the heat-to-electricity conversion efficiency, and the thermoelectric figure of merit should be complemented.

Response to reviewer: We thank the reviewer for the constructive suggestion that helps us to improve our manuscript.

We agree with the reviewer that, for satellite-to-ground communications which require high power solutions as discussed in comment 2, both the heat-to-electricity conversion efficiency and the thermoelectric figure of merit should be more considered for improving the performance. According to the reviewerâs suggestion, we have revised the discussion mentioning only ground-ground communications, and added the corresponding discussions on the detection limitation in relation to the heat-to-electricity conversion efficiency and the thermoelectric figure of merit in the Revised Manuscript, and copied below:

On page 15 of the revised Manuscript.

â¦â¦On the other hand, the response sensitivity of the as-proposed detector should be further enhanced by improving the heat-to-electricity conversion efficiency and the figure of merit of the thermoelectric materialsâ¦â¦

Reviewer comments:

Reviewer #3

(Remarks to the Author)
Thank you for addressing some of my concerns in your revised manuscript. However, I remain unconvinced by the responses provided, and I would like to elaborate on my reasons.

Compared to the state-of-the-art in nanoantenna design, specifically metasurfaces, for SAM and OAM detection, the configuration presented in this work appears quite modest. Although the authors claim that previous efforts were focused on passive devices, this work predominantly relies on similar principles without significant advancements. The incorporation of PET material does introduce active behavior, enabling direct electric readout of OAM signals. Nevertheless, this improvement seems more suited to specialized journals such as Small or Advanced Optical Materials. The versatility and robustness of the PET material, including aspects like low-power resolution and detection limits for shorter wavelengths, have not been adequately discussed. Moreover, a truly active device should depend on an external power source to control or modify the electrical signals, rather than merely enabling straightforward readout. This critical attribute is lacking in the current work.

Additionally, I sought innovation in terms of the working spectrum to assess if the device could bridge technological gaps for new applications. While the authors mention potential applications in earthquake relief, emergency response, and anti-terrorism, these claims are not supported by practical demonstrations.

Open Access This Peer Review File is licensed under a Creative Commons Attribution 4.0 International License, which permits use, sharing, adaptation, distribution and reproduction in any medium or format, as long as you give appropriate credit to the original author(s) and the source, provide a link to the Creative Commons license, and indicate if changes were made.
In cases where reviewers are anonymous, credit should be given to 'Anonymous Referee' and the source.
The images or other third party material in this Peer Review File are included in the article's Creative Commons license, unless indicated otherwise in a credit line to the material. If material is not included in the article's Creative Commons license and your intended use is not permitted by statutory regulation or exceeds the permitted use, you will need to obtain permission directly from the copyright holder.
To view a copy of this license, visit https://creativecommons.org/licenses/by/4.0/
